# PRIMA-1^MET^-induced neuroblastoma cell death is modulated by p53 and mycn through glutathione level

**DOI:** 10.1186/s13046-019-1066-6

**Published:** 2019-02-12

**Authors:** Vid Mlakar, Simona Jurkovic Mlakar, Laurence Lesne, Denis Marino, Komal S. Rathi, John M. Maris, Marc Ansari, Fabienne Gumy-Pause

**Affiliations:** 1 CANSEARCH Research Laboratory, Faculty of Medicine, University of Geneva, Geneva, Switzerland; 2 Division of Oncology and Center for Childhood Cancer Research, Children’s Hospital of Philadelphia, Philadelphia, PA USA; 3 Department of Pediatrics, Perelman School of Medicine at the University of Pennsylvania, Philadelphia, PA USA; 4 Department of Pediatrics and Adolescent Medicine, Onco-Hematology Unit, Geneva University Hospital, Geneva, Switzerland

**Keywords:** Neuroblastoma, PRIMA-1^MET^, p53, MYCN, Glutathione

## Abstract

**Background:**

Neuroblastoma is the most common extracranial solid tumor in children. This cancer has a low frequency of *TP53* mutations and its downstream pathway is usually intact. This study assessed the efficacy of the p53 activator, PRIMA-1^MET^, in inducing neuroblastoma cell death.

**Methods:**

CellTiter 2.0 was used to study susceptibility and specificity of NB cell lines to PRIMA-1^MET^. Real-time PCR and western blot were used to assess the most common p53 transactivation targets. Induction of p53 and Noxa, and inhibition of Cas3/7, were used to assess impact on cell death after PRIMA-1^MET^ treatment. Flow cytometry was used to analyze cell cycle phase and induction of apoptosis, reactive oxygen species, and the collapse of mitochondrial membrane potential.

**Results:**

Neuroblastoma cell lines were at least four times more susceptible to PRIMA-1^MET^ than were primary fibroblasts and keratinocyte cell lines. PRIMA-1^MET^ induced cell death rapidly and in all cell cycle phases. Although PRIMA-1^MET^ activated p53 transactivation activity, p53’s role is likely limited because its main targets remained unaffected, whereas pan-caspase inhibitor demonstrated no ability to prevent cell death. PRIMA-1^MET^ induced oxidative stress and modulated the methionine/cysteine/glutathione axis. Variations of MYCN and p53 modulated intracellular levels of GSH and resulted in increased/decreased sensitivity of PRIMA-1^MET^. PRIMA-1^MET^ inhibited thioredoxin reductase, but the effect of PRIMA-1^MET^ was not altered by thioredoxin inhibition.

**Conclusions:**

PRIMA-1^MET^ could be a promising new agent to treat neuroblastoma because it demonstrated good anti-tumor action. Although p53 is involved in PRIMA-1^MET^-mediated cell death, our results suggest that direct interaction with p53 has a limited role in neuroblastoma but rather acts through modulation of GSH levels.

**Electronic supplementary material:**

The online version of this article (10.1186/s13046-019-1066-6) contains supplementary material, which is available to authorized users.

## Background

Neuroblastoma (NB) is the most common extracranial solid tumor in children. Current event-free survival rates range from 75% to more than 85% for low- and very low-risk groups, to less than 50% for high-risk patients despite dose-intensive therapy [1, 2]. NB progression to an advanced stage and poorer overall survival is characterized by specific molecular events, the most common of which are *MYCN* amplification (MNA) [2, 3] and 11q deletion [4]. NB show a low rate of point mutations, and predominant events leading to tumor progression are chromosomal rearrangements due to apparent chromosomal instabilities [5–8]. Fifty percent of all human cancers contain mutation in the tumor suppressor gene *TP53*, and some estimations contend that almost all cancers evolve a way to circumvent the p53 pathway [9]. NB has been suggested as a good candidate for reactivation of the p53 pathway because it has a low frequency of mutations in *TP53* [10, 11]. The downstream pathway is usually intact, with most of the mutations appearing to be in the upstream MDM2-p14(ARF)-p53 network [12]. Nutlin-3 and its cis-imidazoline analogues activate p53 by inhibiting p53-MDM2 interaction. Preclinical investigation on NB cell lines was encouraging, demonstrating good responses in vitro [11, 13]. In vivo studies in mice suggest that MDM2 inhibitors could be well-tolerated [14]. Clinical trials in liposarcoma patients using Nutlin-3 analogues did not prove effective, however, and revealed an association with severe thrombocytopenia and neutropenia [15]. In addition, resistance can readily develop in cancer cells exposed to selection pressure by selecting cells with *TP53* mutation, which dramatically reduces the efficacy of Nutlin-3 [16].

A new group of molecules that are able to directly activate mutated p53 was recently developed [17, 18]. The most promising, PRIMA-1^MET^, is currently being investigated in several early-stage adult clinical trials (NCT02098343, NCT02999893, NCT03072043, NCT03588078, NCT03745716, NTC03391050, NTC03268382 and NTC00900614). In vivo, PRIMA-1^MET^ is converted into the active compound methylene quinuclidinone (MQ), which reacts with the thiol group of cysteine in proteins. Studies by Lambert et al demonstrated that PRIMA-1^MET^ binds to p53, thus restoring p53 function by refolding the protein in its native structure [18]. In vitro cells and in vivo mouse studies on various cell lines suggest good efficacy of PRIMA-1^MET^ on adenocarcinoma and non-small cell lung cancer [19, 20], colorectal cancer [21], glioblastoma [22], multiple myeloma [23, 24], acute myeloid leukemia [25], breast cancer [26], and ovarian cancer [27] cell lines. Interestingly, depending on the cancer type, PRIMA-1^MET^ induced death was not always p53 dependent. Different off-target effects involving ROS toxicity or autophagy were reported (recently reviewed by Perdrix et al [28]).

This study aimed to evaluate the efficacy of PRIMA-1^MET^ in NB cell lines and to explore the roles of p53, MYCN, glutathione (GSH) and thioredoxin (TXN) systems in PRIMA-1^MET^ efficacy and cellular response to PRIMA-1^MET^.

## Methods

### Cell lines and chemicals

The NB cell lines CHP212, LAN6, NBL-S, NGP, SK-N-DZ and SK-N-SH were provided by Dr. E. Attiyeh and Prof. J. Maris (Children’s Hospital of Philadelphia, Philadelphia, USA). The CLB-GA NB cell line was provided by Dr. V. Combaret (Centre de Ressources Biologiques du Centre Léon Bérard, Lyon, France). BE-(2)C, LA1–55 N, and SK-N-DZ were purchased from ATCC (USA). All NB cell lines were maintained in a standard NB medium composed of DMEM supplemented with 10% FBS, 1% antibiotic/antimycotic solution, and 1% L-glutamine. All NB cell lines passed identity and mycoplasma testing performed independently by Microsynth AG (Switzerland). Human normal primary keratinocytes and fibroblasts (LGC, Germany) were maintained in a dermal cell basal medium supplemented with keratinocyte growth kit and low serum fibroblast basal medium, respectively, prepared according to the manufacturer’s recommendations (LGC, Germany). LCL (lymphoblastoid cell lines, LGC, Germany) were maintained in RPMI 1640 supplemented with 10% FBS and 1% antibiotic/antimycotic solution according to manufacturer’s recommendations. The following compounds were used: PRIMA-1^MET^ (50 mM in H_2_O, Abcam, UK), pan-caspase inhibitor Z-VAD-FMK (25 mM in H_2_O, LubioScience, Switzerland), etoposide (42,4 mM in DMSO), glutathione (100 mM in H_2_O), JQ-1 (1 mM in H_2_O), L-cysteine (50 mM in H_2_O), L-homocysteine (100 mM in H_2_O), L-methionine (12 g/L in H_2_O), N-acetyl-cysteine (100 mM in H_2_O), orlistat (20 mM in DMSO) (Sigma-Aldrich, Germany), buthionine sulfoximine (BSO, 14.4 mM in H_2_O, TRC, Canada). A stably expressing p53 LA1–55 N cell line was obtained by transfection of pcDNA3, containing the p53 gene, using X-tremeGENE HP DNA Transfection Reagent (Roche, Switzerland) and subsequent selection with G418 (Promega, USA) according to the manufacturer’s recommendations. Concentrations of PRIMA-1^MET^ were adjusted for each cell line to obtain precise 50% inhibitory concentration (IC50) values. Vehicle control was used in all experiments.

### Cell survival assay

CellTiter 2.0 (Promega, USA) was used according to the manufacturer’s recommendations to measure the number of live cells. 100 μL of CellTiter 2.0 was added to each well containing 7500 cells treated with PRIMA-1^MET^ for 24 h in 100 μL of medium. Plates were incubated for 2 min on a horizontal shaker at medium speed and at room temperature. Chemiluminescence was measured using Victor3 (Thermo Fisher Scientific, USA) for 1 s, after shaking at medium speed and without the use of filters. Real-time cell viability after treatment with PRIMA-1^MET^ or etoposide and inhibition of caspase by pan-caspase inhibitor was followed using RealTime-Glo MT Cell Viability Assay (G9711, Promega, Madison, USA) according to the manufacturer’s recommendations. Chemiluminescence was measured using SpectraMax iD3 (Molecular Devices, USA) every 10 min for 22 h at 37 °C.

### Flow cytometry

CyAn ADP flow cytometer (Beckman Coulter, USA) was used with the following kits according to manufacturers’ recommendations: FITC Annexin V Apoptosis Detection Kit I (BD Pharmingen, USA), CellROX Oxidative Stress Reagent (Molecular Probes, USA), Autophagy detection kit (Abcam, UK) and mitochondrial JC-1 dye (Invitrogen, USA). Cell cycle, induction of caspase 3 and 7 (Cas3/7), and cellular membrane stability were investigated simultaneously using the FORTESSA flow cytometer system (BD Biosciences, USA). Results were analyzed using Kaluza software (Beckman Coulter, USA).

### In-cell Western blots and Western blots

The intracellular concentration of proteins was measured using CellTag 700 Stain and In-Cell Western (ICW) Assay Kit 1 (LI-COR, USA) according to the manufacturer’s recommendations, 6 h after exposure to 60 μM PRIMA-1^MET^. The following primary mouse antibodies were used for protein labelling. Abcam (UK): Anti-ATM (ab-78), Anti-TXNRD1 (ab16847), Anti-Bax (ab77566) and Anti-Actinβ (ab6276). Cell Signaling Technology (USA): Phospho-p53 (Ser-15) (16G8) (9286), Phospho-ATM (Ser1981) (4526). Santa Cruz (USA): Anti-p53 (sc-126), Anti-p21 (sc-53,870). Antibody specificity was assessed using WB as described in our previous publication [29]. Etoposide, a known inducer of the ATM/p53 pathway [29], was used as a positive control.

### Real-time PCR and gene expression

Real-time PCR was used to measure the expression of *p21*, *14–3-3 (YWHAQ)*, *GADD45*, *PUMA (BBC3)*, *Bax*, *MDM2*, *Noxa (PMAIP1)*, *CXCR4*, and *NMYC*. *GAPDH*, *EEF1A1*, and *TBP* were used as a normalization control (Additional file 2: Table S1). PrimeScript Reverse Transcriptase enzyme (Takara Bio Inc., Japan) and 2x Power SYBR Green Master Mix (Applied Biosystems, USA) was used for generation of cDNA and real-time PCR according to the manufacturer’s recommendations. PCR was performed on an SDS 7900HT instrument (Applied Biosystems, USA). Raw Ct values obtained with SDS 2.2 (Applied Biosystems, USA) were imported in Excel and normalization factor and fold changes were calculated using the GeNorm method [30].

### Luciferase reporter assay

pGL10.4 (Promega, USA) was used to study the impact of PRIMA-1^MET^ on the Noxa promoter region. Three plasmids containing *NOXA* promoter region from + 198 to − 157 were designed. In two of the plasmids, additional mutations were inserted in p53 binding site and CREB binding site. All plasmids were prepared by GenScript (USA). The pRL-SV40 *Renilla* luciferase control reporter vector (Promega, USA) was used as a normalization control at a 1:10 weight ratio. Plasmids were transfected into SK-N-SH cells using X-tremeGENE HP DNA Transfection Reagent (Roche, Switzerland) according to manufacturer’s recommendations. The Dual-Luciferase Reporter Assay System (Promega, USA) and Victor3 (Thermo Fisher Scientific, USA) were used to measure luciferase activity (5 s shaking at medium speed prior to 1 s measurement time without the use of filters).

### Cysteine, glutathione and S-adenozyl-homocysteine concentration, glutathione transferase activity and thioredoxin reductase activity assay

The concentration of total intracellular glutathione (GSH-Glo Glutathione Assay, Promega, USA), cysteine (Cysteine Assay Kit (Fluorometric), Abcam, UK), S-adenozyl-homocysteine (S-Adenosylhomocysteine (SAH) ELISA Combo Kit, Cell Biolabs, USA), glutathione transferase activity (Glutathione S-Transferase (GST) Assay Kit, Sigma Aldrich, USA) and the activity of thioredoxin reductase (Thioredoxin Reductase (TrxR) Assay Kit, Abcam, UK) was measured according to the manufacturer’s recommendations. The Victor3 reader and Versamax microplate reader (Molecular Devices, USA) were used to measure luminescence and absorbance (412 nm), respectively.

### Whole-exome sequencing and bioinformatics

The Illumina HiSeq 2500 sequencer (Illumina, USA) was used for whole exome sequencing according to manufacturer’s recommendations. Illumina paired-end reads were aligned to the human UCSC hg19 reference sequence using BWA software (version 0.7.10). Single-nucleotide variants were called with GATK (version 3.4.46) and annotated with Annovar (version 2016 Feb 01) and SnpEff (version 4.3). Ingenuity variant analysis (QIAgen, Germany) was used to predict the number of cancer driver mutations and to search for *ALK* and *TP53* variants. Affymetrix expression data from GSE3960 were normalized using Robust Multichip Average (RMA) and was used to investigate the association between MNA and expression of genes in the TXN and GSH pathways.

### Statistics

ANOVA *p*-values were used to determine significant differences in expression levels between MNA and non-MNA samples. A t-test was used to determine association of MNA and difference in expression of GCLM versus GCLC genes. A two-way t-test was used to calculate statistical significance for cell-based experiments. Robust nonlinear fit of dose vs. response method (GraphPad Prism 7, USA) was used to calculate the IC50 from relative cell survival, as determined by using CellTiter2.0. The coefficient of drug interaction (CDI) was calculated according to the following formula: CDI = AB/(AxB), where AB represents the relative cell viability for the combination of drugs and A and B represent the relative viability for each compound alone. CDI less than 0.8 was considered indicative of synergy. CDI between 0.8 and 1.2 as additive, while CDI more than 1.2 indicated antagonism [31]. Activity of glutathione S - transferase was calculated using GraphPad Prism 7. If not otherwise noted, measurements were taken in at least triplicate in each experiment, and at least two independent experiments were performed.

## Results

### Genetic characteristics of the eight neuroblastoma cell lines used in this study

The eight NB cell lines used in this study were analyzed for their genetic features (Table 1). *MYCN* amplification and 11q-deletion were detected in four cell lines each. These two features overlap in the cell lines NGP and SK-N-DZ, which were found to have both MNA and 11q-deletion. MNA and 11q-deletion were assessed using real-time PCR (Fig. 3d) and loss of heterozygosity, respectively. The results concur with previous publications, ours and others’ [29, 32, 33]. Mutations in cancer driver genes were detected with whole exome sequencing. The NB cell lines contained an average of 783 cancer-driving variants in an average of 488 different genes (Additional file 1). Three of the cell lines (CLB-GA, LAN6, and SK-N-SH) carry *ALK* variants; the activating mutations F1174 L and R1275Q have been demonstrated to be oncogenic in an NB mouse model [34]. The NGP cell line exhibits high expression of *MDM2* as assessed by real-time PCR. *TP53* mutations were found in three NB cell lines (BE-2C, NGP, and SK-N-DZ). Mutation R110L, found in SK-N-DZ, has been reported in patients with Li-Fraumeni syndrome and results in high expression and abnormal localization of p53, as well as impairment of proper p53 oligomerization [35]. Our results confirm this observation, as SK-N-DZ exhibited high baseline p53 expression (Fig. 3c). The *TP53* mutations found in the NGP cell line, A159D (COSM11496) and C141W (COSM44204), are both deleterious mutations leading to amino acid substitution. No loss of heterozygosity at the *TP53* locus was detected in NGP. This last result suggests that the NGP cell line has no gross deletions at this locus, but it is unknown whether both alleles are inactivated. BE-2C carries the previously described homozygous C135F mutation and has a loss of chromosome 17 at the site of *TP53* [36]. Interestingly, in the three cell lines carrying one or more *TP53* variants, *MYCN* was found to be amplified (Table 1).

**Table 1:** Characteristics of the neuroblastoma cell lines used in this study

Cell line	*MYCN* ^a^	11q^b^	*TP53* status and p53 expression^c^	*MDM2* expression^*d*^	*ALK status* ^c^	Number of cancer driver variants (genes)	Reference
BE-2C	amp	no del	C135F^e^ High expression	–	wt	908 (629)	[29, 50, 51]
CHP212	amp	no del	wt	–	wt	619 (354)	[29, 50]
CLB-GA	wt	no del	wt	–	R1275Q (rs113994087)	715 (394)	[29, 32]
LAN6	wt	del	wt	–	D1091N (rs864309584)	754 (444)	[11, 29, 51]
NBL-S	wt	del	wt	–	wt	755 (434)	[29, 50, 51]
NGP	amp	del	A159D^f^ C141W^f^	High expression	wt	1120 (810)	[29, 50]
SK-N-DZ	amp	del	R110L^e^ (rs11540654) High expression	–	wt	733 (453)	[29, 50]
SK-N-SH	wt	no del	wt	–	F1174 L (rs863225281)	660 (388)	[29, 50]

*amp* amplified, *del* deletion, *no del* no deletion, *wt* wild type

^a^Results obtained from the literature and confirmed by real-time PCR expression of *MYCN*

^b^Results verified with analysis of heterozygosity and/or Fish analysis

^c^Results obtained by whole-exome sequencing and confirmed by the literature

^d^Results obtained by real-time PCR and confirmed by the literature

^e^Homozygous mutation/variant

^f^Heterozygous mutation/variant

### PRIMA-1^MET^ is a potent inducer of neuroblastoma cell death

**Fig. 1:**
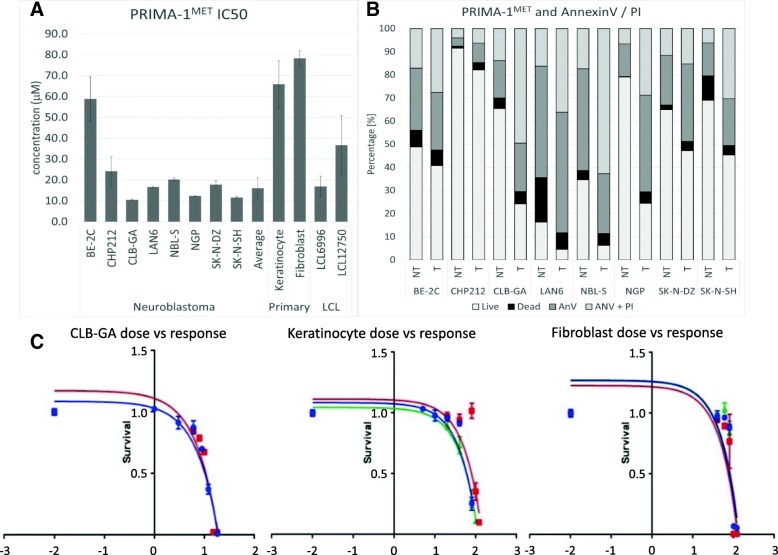
PRIMA-1^MET^ IC50 values. **a**: PRIMA-1^MET^ IC50 values. Eight NB cell lines were tested for PRIMA-1^MET^ IC50 values: BE-2C (58.8 + − 10.72 μM), CHP212 (24.2 + − 7.02 μM), CLB-GA (10.5 + − 0.34 μM), LAN6 (16.5 μM + − 0.48), NBL-S (20.1 + − 0.80 μM), NGP (12.3 + − 0.27 μM), SK-N-DZ (17.8 + − 1.95 μM) and SK-N-SH (11.6 + − 0.55 μM). The average IC50 (excluding resistant BE-2C) for NB cell lines was 16.1 + − 4.99 μM. The PRIMA-1^MET^ IC50 values for the non-tumor cells were: 65.8 + − 11.2 μM (keratinocytes), 78.3 + − 3.69 μM (fibroblasts), 16.9 + − 4.96 μM (LCL6996) and 36.6 + − 14.32 μM (LCL12750). No significant impact of MNA or 11q status on IC50 was observed. **b**: Graph shows increase in dead (black), early apoptotic (dark grey), and late apoptotic (light grey) cells and decrease in live cells (white) 16 h after PRIMA-1^MET^ treatment at IC50 concentration. Treatment with PRIMA-1^MET^ results in significant overall increase in the three populations (dead, early, and late apoptosis) combined and in AnnexinV-positive cells (early and late apoptosis), demonstrating activation of apoptosis after PRIMA-1^MET^. NT – non-treated vehicle control, T – treatment with PRIMA-1^MET^. **c**: The graph shows a non-linear regression fitting for IC50 calculation (BE-2C, keratinocytes, and fibroblasts). The steepness of the curve illustrates PRIMA-1^MET^’s narrow activation window

To investigate the ability of PRIMA-1^MET^ to induce NB cell death, eight NB cell lines were tested to determine IC50 values (Fig. 1a). Seven of the eight cell lines exhibited cell death at a low PRIMA-1^MET^ concentration (mean 16.1 + − 5.02 μM); BE-2C was resistant to high PRIMA-1^MET^ concentrations (58.8 + − 10.72 μM). Next, the cell lines were compared for IC50 and presence of 11q-deletion and MNA, the two most prominent molecular events leading to highly aggressive clinical behavior. No significant difference in IC50 was observed in the subgroupings by these features. However, the two most resistant cell lines (BE-2C and CHP212) have MNA, while the two most sensitive (CLB-GA and SK-N-SH) have normal *MYCN* expression (Fig. 1a). NGP and SK-N-DZ, carrying heterozygous A159D and C141W, and homozygous R110L, respectively, do not differ in IC50 in comparison to the average NB cell line, suggesting these mutations do not affect PRIMA-1^MET^ activity. The BE-2C cell line contains a homozygous substitution of cysteine 135 for phenylalanine, suggesting that C135F could be involved in BE-2C’s increased resistance to PRIMA-1^MET^. To understand whether p53 was associated with IC50 the LA1–55 N cell line, which lacks expression of p53, was transfected with pcDNA3-p53 plasmid. Stably expressing p53 LA1–55 N cells (Fig. 6b) had 1.39-fold higher IC50 values than control LA1–55 N cells transfected with empty pcDNA3 plasmid. IC50 was also investigated in four non-NB cell lines (2 primary and 2 lymphoblast) in order to understand the specificity of PRIMA-1^MET^ to tumor cell lines. Primary keratinocytes and primary fibroblasts were 4.1 times and 4.9 times more resistant, respectively, than the average of the seven sensitive NB cell lines (Fig. 1a). Two immortalized peripheral lymphoblast cell lines (LCL) exhibited significantly lower PRIMA-1^MET^ resistance than both primary cell lines (Fig. 1a). LCL6996 did not demonstrate any significant difference in resistance compared to the average of the NB cells, while LCL12750 was 2.17 times more resistant than LCL6996 (Fig. 1a). This result suggests that Epstein-Barr immortalization, as well as normal population polymorphisms, could affect resistance to PRIMA-1^MET^. Analyzing the dose-response relationship demonstrated that PRIMA-1^MET^ exhibits rapid killing and a narrow activation interval (Fig. 1c). Once the threshold concentration is exceeded, complete cell death is observed within 15 h (Additional file 2: Data for Figure S1).

### PRIMA-1^MET^ induces accumulation of cells in the sub-G1 phase and triggers apoptosis irrespective of cell cycle phase through induction of caspase 3 and 7

**Fig. 2:**
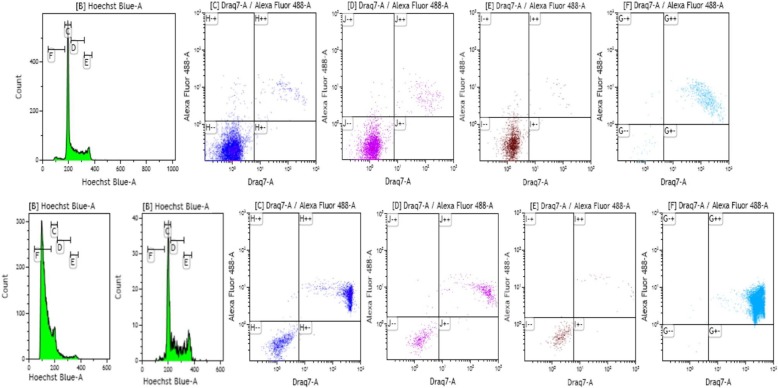
Cell cycle analysis of the NGP neuroblastoma cell line. Top row: non-treated NGP cell line. Bottom row: treated (20 μM PRIMA-1^MET^) NGP cell line. Gates: Green: cell cycle (20 μM Hoechst) **c**: G1 phase (dark blue); **d**: S phase (purple); **e**: G2/M phase (brown); **f**: sub-G1 phase (light blue). Cells in gates C, D, E, and F are distributed according to DRAQ7 and Cas3/7 (AlexaFluor 488) intensity. −/− live cells with stable membrane; −/+ live cells with triggered Cas3/7; +/+ dead cells with triggered Cas3/7 and nonfunctional membrane; +/− dead cells without activated Cas3/7 but with nonfunctional membrane. Results demonstrate that cells treated with PRIMA-1^MET^ die in all phases of the cell cycle and that PRIMA-1^MET^ activates apoptosis with similar intensity to non-treated control cells undergoing apoptosis

To understand how PRIMA-1^MET^ affects NB cell proliferation, cell cycle analysis was performed on the seven sensitive NB cell lines (CHP212, CLB-GA, LAN6, NBL-S, NGP, SK-N-DZ and SK-N-SH). A 20 μM concentration of PRIMA-1^MET^ was used to induce partial cell death, allowing us to analyze the cell cycle after a 24-h exposure. The results show that PRIMA-1^MET^ induces the accumulation of cells in the sub-G1 phase (Fig. 2 and Additional file 2: Data for Figure S2). Simultaneous measurement of Cas3/7 activity and membrane integrity showed that the majority of cells in sub-G1 had significantly increased Cas3/7 activity and compromised membranes (Fig. 2). This phenotype is typical for apoptotic cells; necrotic cells usually do not express high levels of Cas3/7. Results on the cells that survived PRIMA-1^MET^ treatment demonstrate that the cell cycle does not change substantially (Fig. 2, bottom row). Analyzing the Cas3/7 activity and membrane integrity of cells in the G1, S, and G2/M phases revealed that cells undergo apoptosis in all three phases of the cell cycle (Fig. 2 and Additional file 2: Data for Figure S2).

To corroborate this observation, we tested for early stage markers of apoptosis, AnnexinV/PI and collapse of mitochondrial membrane potential. The results showed a significant increase in AnnexinV positive cells, indicating early and late apoptosis after PRIMA-1^MET^ treatment (Fig. 1b). This is supported by results using JC-1 labeling that demonstrate previously described “ballooning” of mitochondria [37] which leads to their subsequent collapse and induction of apoptosis (Additional file 2: Data for Figure S2).

Overall results demonstrate that PRIMA-1^MET^ efficiently kills tumor cells irrespective of the cell cycle phase by triggering apoptosis, as measured by increased Cas3/7 activity and increased levels of AnnexinV. This result is not surprising, considering that most cells die within 15 h of sufficient exposure to PRIMA-1^MET^.

### PRIMA-1^MET^ increases Noxa and decreases CXCR4

**Fig. 3:**
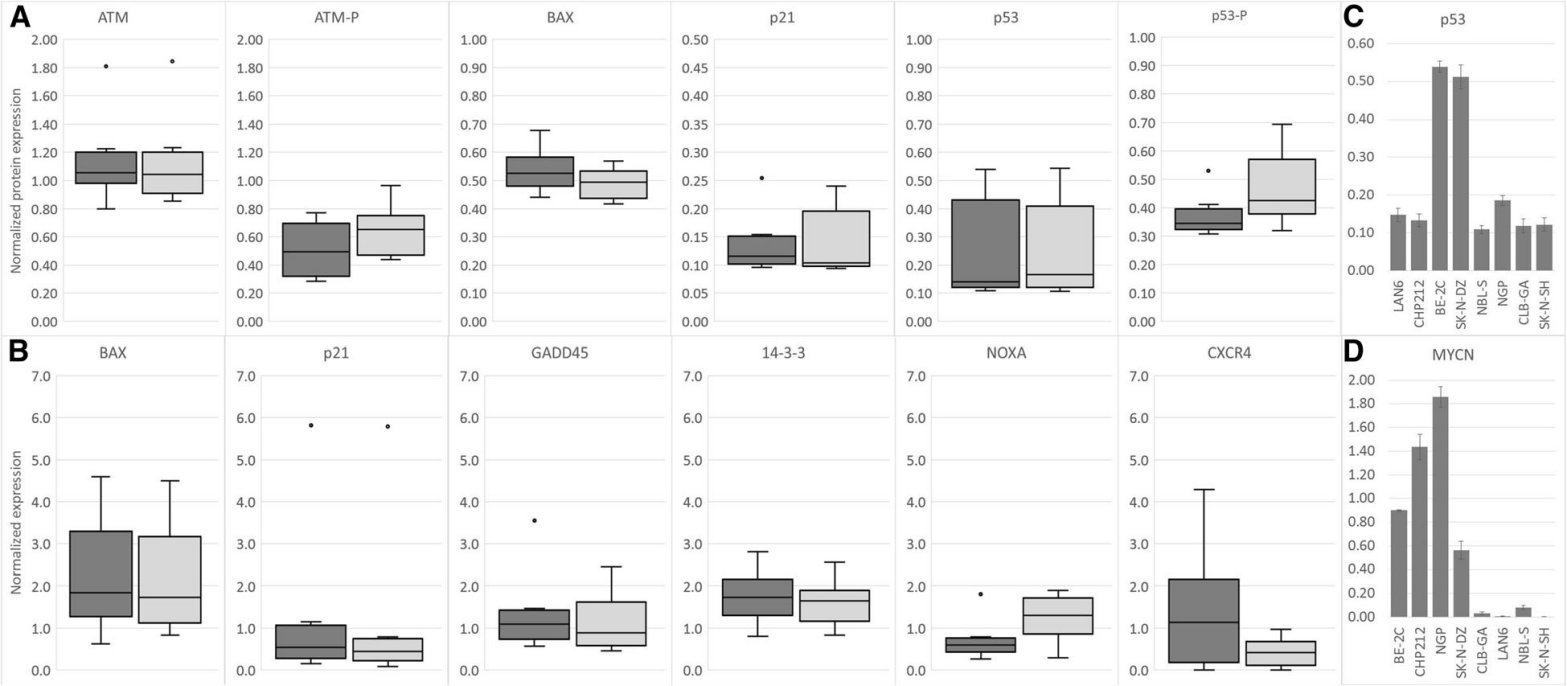
Protein and gene expression after treatment with PRIMA-1^MET^. **a**: Results of protein expression measured by ICW demonstrate that 6 h of 60 μM PRIMA-1^MET^ does not induce phosphorylation of ATM or p53 in NB cell lines (dark grey: non-treated vehicle control, light grey: PRIMA-1^MET^, dots: outliers). There is no significant upregulation of p21 or Bax due to PRIMA-1^MET^ treatment. **b**: Results of gene expression measured by real-time PCR demonstrate that major p53 target genes involved in cell cycle arrest (14–3-3, Gadd45, p21) remain unchanged (dark grey: non-treated vehicle control, light grey: PRIMA-1^MET^, dots: outliers). Bax is involved in signaling apoptosis and did not show any changes in levels as measured by ICW (A) or real-time PCR (B). Noxa was consistently and significantly upregulated and showed on average a 2-fold increase. CXCR4, a marker of aggressive NB, was downregulated after PRIMA-1^MET^ treatment. **c**: BE-2C and SK-N-DZ (both MNA cell lines) showed accumulation of *TP53* mRNA. **d**: BE-2C, CHP212, NGP and SK-N-DZ have high expression of *MYCN*, which is in accordance with the previously-described presence of MNA in these cell lines

Studies performed to date demonstrate that PRIMA-1^MET^ acts by covalently binding to wild-type or mutant p53 and restoring its function, driving cancer cells to apoptosis [18, 38]. To further explore this process, an ICW assay and WB were used to investigate phosphorylation of p53 and ATM and induction of the downstream targets Bax and p21. Concentrations higher than IC50 were used in all subsequent experiments because they reflect better in vivo conditions. Mice tolerated PRIMA-1^MET^ concentrations of up to 100 mg/kg (500 mmol/kg) without apparent side effects [17] while in clinical trials peak concentrations up to 82.6 mg/L (415 μmol/L) demonstrated mild but reversible side effects [39]. Results show that 60 μM PRIMA-1^MET^ does not induce phosphorylation of either ATM or p53 (Fig. 3a). Likewise, no change was detected in the basal levels of ATM, p53, p21, or Bax (Fig. 3a). The only exception to these results was the NGP cell line, in which PRIMA-1^MET^ treatment induced phosphorylation of ATM and p53, as well as overexpression of p21 (data not shown). Investigating the induction of reactive oxidative species (ROS) after PRIMA-1^MET^ treatment in the NGP cell line revealed an increased presence of ROS (Additional file 2: Supplementary data 7), which could explain the induction of ATM, p53, and p21 due to DNA damage. WB was performed on BE-2C and SK-N-DZ to control for the accuracy of the ICW method. Results using WB demonstrate complete concordance of protein expression, as there was no difference after PRIMA-1^MET^ treatment. Etoposide, a positive control, induced expression of all tested proteins (Additional file 2: Data for Figure S3).

Even though we did not observe a change in p53 phosphorylation in our experiments, it has been demonstrated that treatment with PRIMA-1^MET^ is able to restore the p53 transactivation function and signal apoptosis without p53 phosphorylation in the p53-null Saos-2 cell line [17, 18]. It was also reported that, after exposure to PRIMA-1^MET^, Bax could be triggered in p53^WT^ and Noxa in mutant p53^R175H^ cell lines [18]. In our experiments, we observed no change in expression of Bax as measured by ICW and real-time PCR (Fig. 3a and b). Because Bax works through translocation and destabilization of mitochondria, increased expression might not be a prerequisite for its action. Analysis of Bax by immunofluorescence did not demonstrate any change in its localization (data not shown), suggesting that, in NB cell lines, the Bax pathway is not involved in PRIMA-1^MET^-induced apoptosis. PUMA, GADD45, 14–3-3, and Noxa were the other p53 transcriptional targets investigated using real-time PCR. Results demonstrate that Noxa is consistently upregulated after PRIMA-1^MET^ treatment, while GADD45 and 14–3-3 show no change (Fig. 3b). PUMA expression was below reliable detection range in the eight NB cell lines we tested (data not shown). Lastly, CXCR4, a marker of NB aggressiveness and metastasis, and a target for p53 transactivation, was consistently downregulated after PRIMA-1^MET^ treatment, suggesting a potential benefit of PRIMA-1^MET^ in inhibiting tumor spread (Fig. 3B).

### Noxa expression correlates with IC50 and is upregulated through p53

**Fig. 4:**
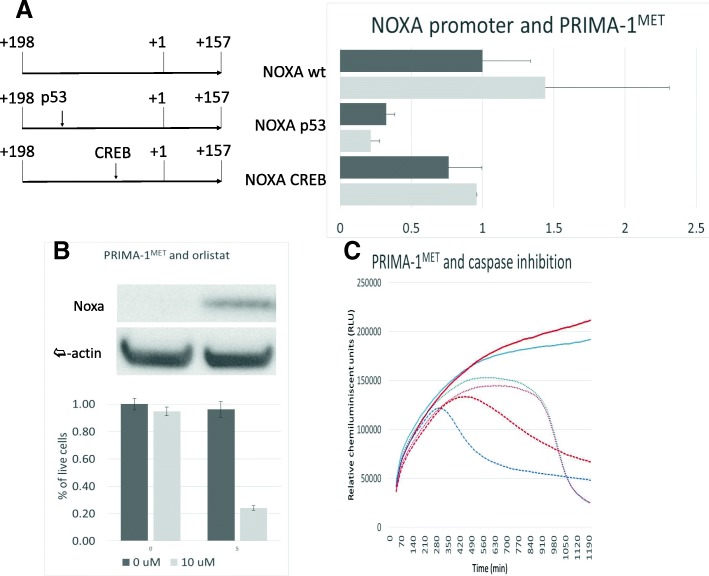
PRIMA-1^MET^ and Noxa. **a**: Results of luciferase reporter assay. A significant increase in luciferase activity after PRIMA-1^MET^ treatment in Noxa wt and Noxa CREB but not Noxa p53 pGL10.4 plasmids. Dark grey: non-treated vehicle control. Light grey: PRIMA-1^MET^ treatment (24 h, 15 μM). NOXA wt: pGL10.4 plasmid with normal Noxa promoter sequence. Noxa p53: pGL10.4 plasmid with Noxa promoter sequence containing mutation at the p53 binding site. Noxa CREB: pGL10.4 plasmid with Noxa promoter sequence containing mutation at the CREB binding site. **b**: Sensitization of SK-N-SH cells by pretreatment with orlistat. Dark grey: non-treated vehicle control. Light grey: PRIMA-1^MET^ treatment (24 h, 10 μM). Left columns: orlistat non-treated vehicle control. Right columns: orlistat treatment (5 μM, 6 h prior to PRIMA-1^MET^ treatment). **c**: Impact of pan-caspase inhibitor on the growth curve of SK-N-SH cells. Red lines: cells treated with pan-caspase inhibitor (22 h, 50 μM). Blue lines: Non-treated vehicle controls. Full line: non-treated vehicle control for PRIMA-1^MET^. Dotted line: PRIMA-1^MET^ treated cell line (22 h, 20 μM). Dashed line: etoposide treatment (22 h, 30 μM)

Saha et al recently reported that, in multiple myeloma cells, Noxa is involved in apoptosis triggered by PRIMA-1^MET^ [23]. In our NB cell lines, a significant correlation (r^2^ = 0.59, *p* = 0.026) was demonstrated between IC50 and PRIMA-1^MET^-induced Noxa expression, suggesting that together with mitochondrial dysfunction (Additional file 2: Data for Figure S2), Noxa could play a role in PRIMA-1^MET^-induced NB apoptosis. Unchanged Noxa expression in the resistant cell line BE-2C, which carries a *TP53* C135 mutation, further supports the involvement of p53. However, other classical p53 targets did not show any induction after PRIMA-1^MET^, thus necessitating investigation of the relationship between p53 and Noxa after PRIMA-1^MET^ treatment. Experiments using the SK-N-SH cell line transfected with plasmids containing mutations in p53 or CREB binding segments of the Noxa promoter demonstrate 3.1-fold (*p* < 0.0001) or 1.3-fold (*p* < 0.026) decrease in activity when the p53 or CREB site is mutated, respectively (Fig. 4a). When transfected SK-N-SH cells were exposed to PRIMA-1^MET^, we observed significant induction of luciferase in cells with wt (1.44-fold, *p* = 0.03) and CREB-mutated (1.26-fold, *p* = 0.025) plasmids, but no significant difference in cells with plasmids containing a p53 binding site mutation (Fig. 4a). To control for p53 action on this particular site, cells with wt and p53-mutated plasmids were treated with etoposide, a known inducer of p53. Similarly, significant luciferase induction was observed in SK-N-SH cells containing wt plasmid (1.86-fold, *p* = 0.007) and no change was observed in SK-N-SH cells bearing plasmid with p53 binding site mutation (0.92-fold, *p* = 0.07) (Additional file 2: Data for Figure S4A). Although Noxa gene expression was clearly up-regulated after PRIMA-1^MET^ treatment, we were unable to demonstrate an increase in Noxa protein expression in all eight of the NB cell lines tested, either before or after PRIMA-1^MET^ treatment, because Noxa protein levels were not high enough for detection using WB. Next, Orlistat, a known Noxa stabilizer was used to increase Noxa protein expression. While no significant difference was detected in cell survival after treatment with orlistat alone, a significant decrease in live cells was observed when PRIMA-1^MET^ was added (Fig. 4b). To address whether inhibition of general apoptosis, of which Noxa is an essential part, is enough to inhibit death induced by PRIMA-1^MET^, pan-caspase inhibitor was used. Our results demonstrate a partial but significant rescue of SK-N-SH cells from apoptosis induced by etoposide, used as positive control, but not from death induced by PRIMA-1^MET^ (Fig. 4c).

### PRIMA-1^MET^, GSH pathway and p53

**Fig. 5:**
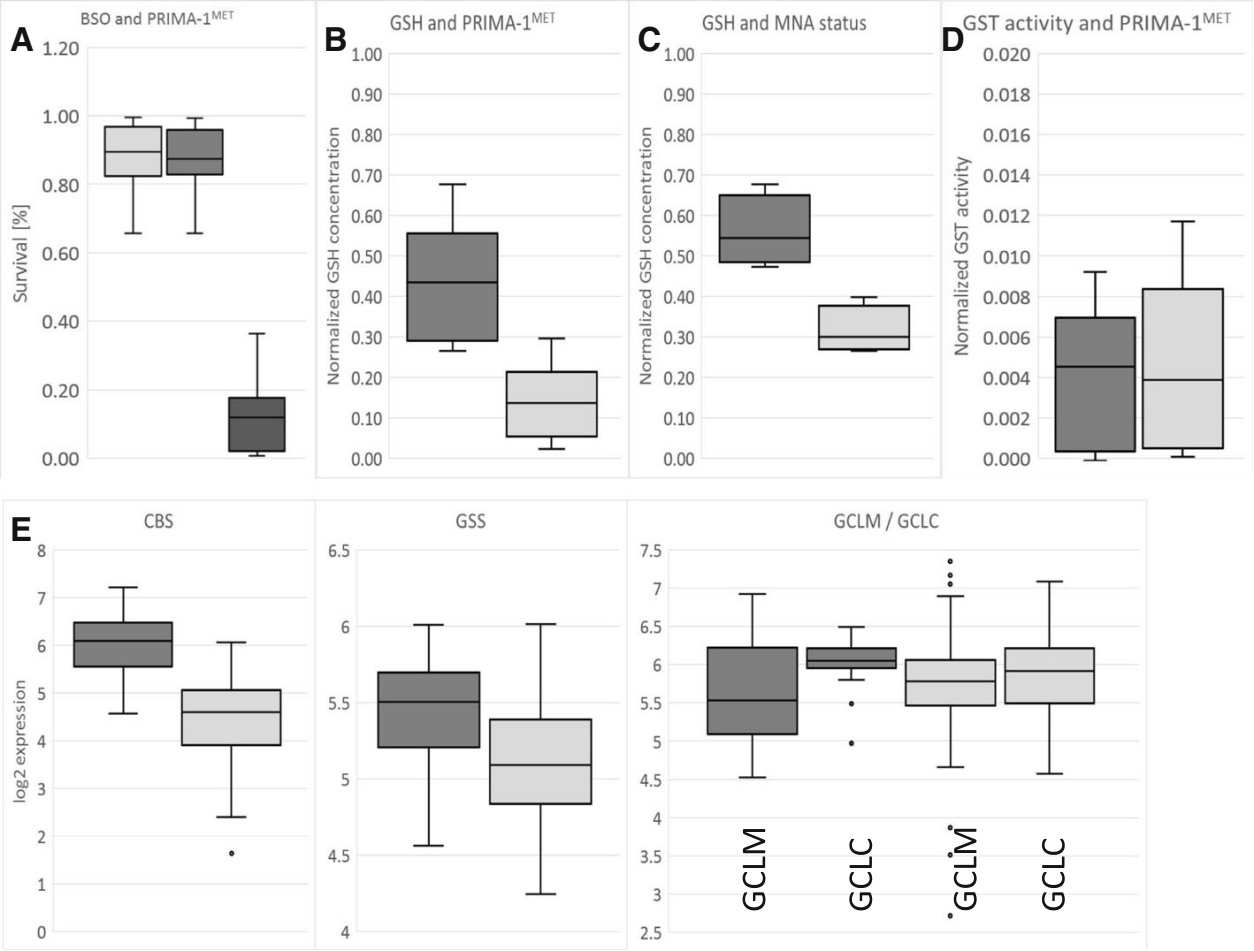
PRIMA-1^MET^ and glutathione. **a**: Concurrent BSO and PRIMA-1^MET^ treatment. All cell lines were treated with fixed 100 μM BSO for 6 h prior to PRIMA-1^MET^ treatment at a concentration of less than IC90 on average. The Y-axis indicates the survival ratio. A significant synergistic effect (CDI = 0.16) due to BSO treatment was detected in all cell lines, indicating that the depletion of GSH significantly influences the sensitivity of the cells to PRIMA-1^MET^. Light grey: survival at 100 μM BSO, dark grey: survival at IC90 concentration of PRIMA-1^MET^, black: BSO and PRIMA-1^MET^. **b**: Concentration of GSH decreases 3.1-fold (*p* < 0.001) in NB cell lines treated with 60 μM PRIMA-1^MET^ for 6 h (light grey) in comparison to non-treated (dark grey). **c**: Concentration of GSH is 1.8-fold (*p* < 0.05) lower in MNA NB (dark grey) in comparison to non-MNA NB (light grey). **d**: No significant difference in GST activity due to PRIMA-1^MET^ (light grey) in comparison to non-treated control (dark grey) NB cell lines. **e**: Higher expression of CBS (2.97-fold, p < 0.001) and GSS (1.25-fold, p < 0.001), and significant change in GCLC/GCLM ratio (1.3-fold, *p* = 0.037) in MNA NB samples (dark grey) in comparison to non-MNA NB samples (light grey). Dots: outliers

GSH plays a central role in cellular redox control and in the xenobiotic neutralizing system. It has been reported that inhibiting GSH with BSO sensitizes lung cancer cells to PRIMA-1^MET^ [18] by decreasing the intracellular concentration of GSH. To investigate if the same mechanism works in NB, a combinatorial treatment of BSO and PRIMA-1^MET^ was tested. BSO treatment alone has a limited short-term effect on NB cell survival. After 30 h of BSO treatment, only SK-N-DZ and NGP had less than 80% survival. All the NB cells, including p53-null LA1–55 N, treated with 100 μM of BSO 6 h prior to 24 h treatment with PRIMA-1^MET^ were highly sensitized to PRIMA-1^MET^ (Fig. 5a and Additional file 2: Data for Figure S5A). To understand how the intracellular concentration of GSH is affected by PRIMA-1^MET^, it was measured 8 h after exposure to PRIMA-1^MET^. A significant decrease (3.1-fold on average) of GSH concentration was observed in all eight of the NB cell lines (Fig. 5b), including in p53-null LA1–55 N (Additional file 2: Data for Figure S5A). Because this result suggested an alteration of the methionine/cysteine/GSH axis, we investigated whether supplementation of cysteine-related metabolites (GSH, N-acetyl-cysteine, cysteine, homocysteine, and methionine) was able to reverse the action of PRIMA-1^MET^ (Additional file 2: Data for Figure S5B). The results of co-treatments demonstrated that 400 μM of either N-acetyl-cysteine, cysteine, homocysteine, or GSH were able to reverse the effect of PRIMA-1^MET^ completely, but 400 μM of methionine could not do so in any of the six different NB cells, including p53-null LA1–55 N (Additional file 2: Data for Figure S5B). A complete reversal effect was possible, even after prolonged exposure to PRIMA-1^MET^, immediately before cell death, as indicated by profound morphological changes and complete reversal of autophagy (Additional file 2: Data for Figure S5D). To understand where possible inhibition of the methionine/cysteine/GSH axis might occur, we measured concentrations of the pathway’s main metabolites (S-adenozyl-methionine (SAH) and cysteine) in the cells, 1 h and 11 h after PRIMA-1^MET^ exposure (Additional file 2: Data for Figure S5C). Results indicated that treatment using PRIMA-1^MET^ significantly reduced SAH concentrations (0.71-fold and 0.66-fold at 1 h and 11 h, respectively) but increased cysteine concentrations (1.27-fold at 11 h), whereas the cysteine/SAH ratio had increased by 1.56-fold at 1 h to 1.92-fold at 11 h (Additional file 2: Data for Figure S5C). These results demonstrated that GSH was an important defense mechanism against PRIMA-1^MET^ that was modulated by PRIMA-1^MET^.

### P53 and MNA status affects concentration of GSH and modulates resistance of neuroblastoma cells against PRIMA-1^MET^

**Fig. 6:**
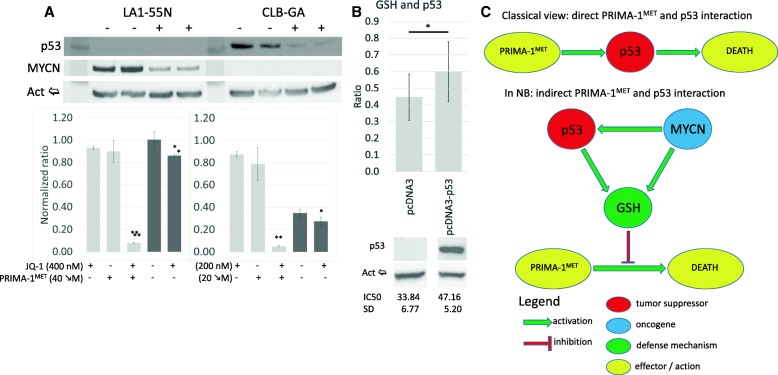
PRIMA-1^MET^, p53 and MYCN. **a**: Inhibition of MYCN and p53 expression by JQ-1. LA1–55 N (p53-null) cells showed a significant decrease in MYCN, whereas CLB-GA (non-MNA) cells demonstrated almost complete suppression of p53 expression after 16 h treatment. - – Non-treatment control, + − 16 h, 200 nM JQ-1. PRIMA-1^MET^ and JQ-1 demonstrated synergism in both cell lines (CDI_LA1–55N_ = 0.1, CDI_CLB-GA_ = 0.07). Treatment with JQ-1 resulted in a significant decrease in the concentration of GSH in LA1–55 N (1.163-fold, *p* < 0.01) and CLB-GA (1.26-fold, *p* = 0.028). Light grey – JQ-1/PRIMA-1^MET^ co-treatment, dark grey – GSH concentration after JQ-1 treatment, *p < 0.05, **p < 0.01. **b**: Concentrations of GSH and IC50 in LA1–55 cells with and without p53 gene expression. Results demonstrated 1.34-fold (*p* = 0.029) increase in total cellular GSH and 1.39-fold (*p* = 0.007) IC50 increase in cells expressing p53. Upper – concentration of GSH normalized to the number of cells using CellTiter2.0. Middle – WB for p53. Bottom – IC50 values for cells with and without p53. * - *p* < 0.05. **c**: New proposed model of interaction between PRIMA-1^MET^, p53, and MYCN

Because MNA is one of the main genomic events in NB, we investigated MNA’s impact on GSH. Before PRIMA-1^MET^ treatment, GSH concentration correlates with MNA, but not with IC50 or 11q-deletion. MNA cells have on average 1.8 times (*p* < 0.05) more GSH (Fig. 5c). The decrease in GSH concentration due to PRIMA-1^MET^ was not significantly associated with MNA, 11q-deletion, or IC50 (data not shown). To investigate how GSH levels differ between MNA and non-MNA NB, correlation between *MYCN* status and gene expression of the main enzymes involved in GSH synthesis was analyzed using the Affymetrix expression array dataset consisting of 101 NB primary tumors (GEO accession: GSE3960). The results demonstrate that expression of *CBS* and *GSS*, genes involved in cystathionine and glutathione synthesis, are 2.97 times (*p* < 0.001) and 1.25 times (p < 0.001) higher, respectively, in MNA than in non-MNA tumors (Fig. 5e). GCLC and GCLM, which form a heterodimeric enzyme, are responsible for the synthesis of γ-glutamyl-cysteine. In MNA NB tumors, expression of *GCLC* (the catalytic subunit) is 1.3 times higher (p < 0.05) than *GCLM* expression, but there was no difference in non-MNA NB tumors (Fig. 5e). The other components of GSH metabolism (*GSTM1, GSTM2, GSTM3, GSTA1, GSTT1*) were also investigated in order to understand the potential influence of MNA on GSH, but no significant differences were detected between MNA and non-MNA NB tumors (data not shown). Finally, to explore the interaction between PRIMA-1^MET^ and the glutathione-S-transferase (GST) system, we measured overall GST activity in the eight NB cell lines. No significant change was observed after PRIMA-1^MET^ treatment (Fig. 5d). MNA’s involvement in the resistance of neuroblastoma cells was explored using JQ-1, a known inhibitor of MYCN expression [40]. Results using JQ-1 demonstrated a simultaneous decrease in MYCN expression and GSH levels (1.16-fold, *p* < 0.01) and sensitized p53-null LA1–55 N cells to higher doses of PRIMA-1^MET^ (Fig. 6a). In contrast, expressing p53 in LA1–55 N resulted in a significant 1.34-fold increase in GSH and a 1.39-fold (*p* = 0.007) increase in IC50 (Fig. 6b). Because MYCN is a known transcription factor of p53 and JQ-1 is known to reduce p53 [40], we investigated p53 expression in CLB-GA (non-MNA, normal p53) cells after exposure to 200 nM JQ-1. JQ-1-treated CLB-GA cells demonstrated a marked decrease in p53 expression, no change in MYCN levels, a significant decrease in GSH levels (1.26-fold, *p* = 0.028), and increased susceptibility to higher doses of PRIMA-1^MET^ (Fig. 6a). Cumulatively, these results demonstrated that MYCN and p53 are implicated in the survival of cells exposed to PRIMA-1^MET^ via the modulation of GSH levels.

### PRIMA-1^MET^ inhibits thioredoxin reductase activity in neuroblastoma cell lines

Another system potentially implicated in resistance to PRIMA-1^MET^ is thioredoxin (TXN). Recently, Peng et al demonstrated that, in lung adenocarcinoma, osteosarcoma, and Burkitt lymphoma cells, PRIMA-1^MET^ can also target thioredoxin reductase 1 (TXNRD1) by binding to its active site, causing a decrease in reductase activity. Interestingly, it was demonstrated that even when inhibited by PRIMA-1^MET^, TXNRD1 is still functional as a pro-oxidant NADPH oxidase. This kind of modulation of TXNRD1 can cause an increase in oxidative stress and cell death, but implications for NB cell survival are questionable [41]. To understand how PRIMA-1^MET^ might influence the TXN system in NB cells, we first used ICW to measure intracellular TXN levels. The results demonstrate that PRIMA-1^MET^ treatment induces no change in total TXN levels in NB cell lines (Additional file 2: Supplementary data 6C). Comparing TXN levels to MNA and 11q status of the cells showed that high levels of TXN are significantly associated with the presence of MNA (Additional file 2: Supplementary data 6B) but not with 11q-deletion. MNA was correlated with 1.2-fold (*p* < 0.02) and 1.22-fold (*p* = 0.02) upregulation in respective expression of *TXN* and *TXN2* in NB tumoral samples. However, no difference in *TXNRD1* expression was observed between MNA and non-MNA NB tumors (Additional file 2: Supplementary data 6E). Because TXN reduction capacity is dependent on the activity of TXNRD1, we measured reductase activity in NB cells. Results demonstrate a significant drop in TXNRD1 activity in all eight NB cell lines (Additional file 2: Supplementary data 6D). No correlation was observed between the reduction rate of TXNRD1 activity and MNA, 11q status, or IC50. BE-2C and SK-N-DZ, two cell lines with aberrant p53 accumulation, demonstrated the smallest inhibition of TXNRD1 activity, and cell lines with *TP53* mutations showed the highest TXNRD1 activity (data not shown). Finally, to address the possible role of the TXN system in resistance to PRIMA-1^MET^, cells were treated concurrently with PX-12 (a known TXN inhibitor) and PRIMA-1^MET^, and survival was analyzed. The results showed an additive effect of PX-12 and PRIMA-1^MET^, but no synergy was observed in any of the eight NB cell lines tested (data not shown). PX-12 alone at 5 μM exhibited the ability to inhibit growth in all NB cell lines, particularly those with MNA (0.54-fold growth compared to 0.82-fold growth in non-MNA lines, *p* = 0.016) (Additional file 2: Supplementary data 6A). This finding could be explained by the higher basal level of TXN in MNA cells (Additional file 2: Supplementary data 6B). An additive effect observed with PX-12 and PRIMA-1^MET^ suggests that PRIMA-1^MET^ action is independent of TXN status. On the other hand, PRIMA-1^MET^ was found to interact with the TXN system through inhibition of TXNRD1, suggesting that drugs interacting with the TXN system might synergize or antagonize with PRIMA-1^MET^.

### PRIMA-1^MET^ and induction of oxidative stress in neuroblastoma cell lines

Because PRIMA-1^MET^ interacts with the GSH and TXN systems, we hypothesized that it could induce oxidative stress in NB. The eight NB cell lines were treated with 60 μM and 120 μM concentrations of PRIMA-1^MET^ for 6 h to understand how PRIMA-1^MET^ could affect the generation of reactive oxygen species in NBs. Higher concentrations were used because they better reflect the physiological reality. Flow cytometry results demonstrate that PRIMA-1^MET^ induced oxidative stress in three cell lines (LAN6, NGP, SK-N-DZ) at 60 μM and in two additional cell lines (BE-2C, CH212) at 120 μM (Additional file 2: Supplementary data 7). Induction of oxidative stress in NGP by PRIMA-1^MET^ could explain ATM- and p53-phosphorylation and p21 overexpression because ROS could lead to an increase in DNA damage. Partially supporting results obtained in other cancer cell lines [18, 41, 42], our data demonstrate that PRIMA-1^MET^ has the ability to induce oxidative stress in NB cell lines. However, this effect is specific to concentration and cell line. Acquired or germline genetic variations might play an important role in the development of oxidative stress due to PRIMA-1^MET^ treatment.

## Discussion

In this study, we have explored the efficacy of PRIMA-1^MET^ in eight NB cell lines and investigated the possible molecular pathways involved in its action and resistance. The data demonstrate that PRIMA-1^MET^ is effective and particularly active in seven of the eight NB cell lines tested. PRIMA-1^MET^ exhibited a narrow activation range, and killed NB cells within 15 h of exposure to IC90 concentration. Unlike initial reports about PRIMA-1^MET^ [18], we demonstrated that the main effect of p53 in NB cells occurs at the level of GSH and not through direct activation of the main p53 targets (Fig. 6). MYCN, which is an important factor in the development of NB, also activates the GSH pathway, resulting in increased levels of GSH and resistance to PRIMA-1^MET^.

PRIMA-1^MET^ is known to restore p53 function after conversion to MQ, a Michael acceptor, which can bind covalently to cysteine and lead to p53 refolding to its native state and inducing cell death [18]. It was therefore surprising that BE-2C, which carries the *TP53* C135F homozygous mutation, was highly resistant. On the other hand, stable expression of p53 in LA1–55 N cells, which otherwise lack p53 expression, increased LA1–55 N resistance to PRIMA-1^MET^. To address these discrepancies, we comprehensively analyzed current evidence about PRIMA-1^MET^ in the context of NB cells. We confirmed that PRIMA-1^MET^ is a potent inducer of apoptosis by analyzing several key markers of apoptosis, namely, induction of AnnexinV, end-stage Cas3/7, perforation of cellular membranes and damage to mitochondria. Our results demonstrate that PRIMA-1^MET^ does not affect the cell cycle, as evidenced by cellular DNA content and low p21 levels. Furthermore, it can induce apoptosis in NB cell lines irrespective of cell cycle phase. This property of PRIMA-1^MET^ could be particularly beneficial since chemotherapeutics such as cisplatin and etoposide usually induce DNA damage followed by cell cycle arrest [43], which leads to the development of resistance and cancer cell survival [7]. It is known that p21, the primary mediator of p53 and a potent cyclin-dependent kinase inhibitor, plays a dual role: it promotes cell cycle arrest during the G1 phase and inhibits proliferation, but it can also promote cell survival by inhibiting apoptosis and giving cells time to repair damaged DNA [44, 45].

Next, the p53 transactivation function was analyzed in detail. Results show no changes in p53, phosphorylated p53, ATM, phosphorylated ATM, or Bax expression levels after PRIMA-1^MET^ treatment. Furthermore, the absence of ATM- and p53-phosphorylation is significant because it demonstrates indirectly that PRIMA-1^MET^ is probably not a DNA-damaging agent, as the ATM/p53 pathway itself is not triggered. Testing for p53 transactivation function showed no induction of many of its major targets, including GADD45, 14–3-3, p21, and Bax. The only exception is Noxa, which is upregulated 2-fold on average. Despite indirect evidence such as Noxa gene expression and IC50 correlation, mitochondrial membrane potential collapse and activation of apoptotic cascade, these results still raised doubts about the direct relationship between PRIMA-1^MET^, p53 and Noxa in NB cell lines. Using luciferase reporter assays, we demonstrated that Noxa upregulation is due to p53, confirming p53 transactivation after PRIMA-1^MET^ treatment. Further investigation of the role of Noxa, using WB and pan-caspase inhibitor, demonstrated that Noxa was not present in NB cells, whereas the inhibition of apoptosis was insufficient to rescue cells from death, thus questioning Noxa’s involvement in PRIMA-1^MET^-mediated death. Nevertheless, Noxa may still prove useful, either as a drug target for Orlistat, which sensitizes cells to PRIMA-1^MET^, or as a marker of PRIMA-1^MET^ potency.

Next, we investigated the role of cellular redox pathways in PRIMA-1^MET^ activity, because PRIMA-1^MET^ is cleared through GSH [18] and influences TXNRD1 reductase activity [41]. Our results demonstrated significant synergy between PRIMA-1^MET^ and BSO, providing strong evidence of GSH but not GST involvement in clearing PRIMA-1^MET^ in NB. Further investigations of the methionine/cysteine/GSH axis demonstrated that metabolites carrying free SH-groups were involved in the inhibition of PRIMA-1^MET^, which leads to a fast reversal of morphology and autophagy. However, the same 400 μM concentration of methionine was unable to rescue the cells. Analysis of intracellular concentrations of cysteine and SAH demonstrated that most of the inhibition of GSH-synthesis by PRIMA-1^MET^ likely happens after cysteine, as its concentration is higher even though the concentration of SAH decreases. Finally, investigating GSH concentration in the context of p53 expression demonstrated an increase of GSH in p53-competent cells, which is in accordance with a recent report by Tarangelo et al [46]. These results suggest that indirect interaction between p53 and PRIMA-1^MET^, through the modulation of GSH, is more relevant than the direct p53 transactivation activity observed in the context of NB (Fig. 6c). Likewise, our results showed that MNA was associated with higher levels of GSH via the increased expression of enzymes associated with GSH synthesis. Further experiments on p53-null LA1–55 N confirmed MYCNs’ independent role in the reduction of GSH levels and increased sensitivity to PRIMA-1^MET^.

In exploring the involvement of the TXN system in PRIMA-1^MET^ efficacy, we found that TXN does not appear to be important for PRIMA-1^MET^ action but that PRIMA-1^MET^ is able to induce TXNRD1 inhibition. It is interesting that PX-12 on its own exhibits a significant effect on NB cell viability, particularly in NB cell lines without MNA. This is probably due to lower gene and subsequent TXN protein expression in comparison to MNA NB. Interestingly, in contrast to previous studies [18, 41, 42], our results suggest that induction of ROS by PRIMA-1^MET^ is frequent but not a universal event and instead depends on molecular context. Interestingly, despite the induction of ROS inactivity of ATM/p53 pathway suggests indirectly that, in NB cell lines, PRIMA-1^MET^ is not involved in DNA damage.

Finally, we found that *CXCR4*, which has been reported to promote NB tumor growth, metastasis and resistance to therapeutics [47, 48] and was therefore chosen in this study as a marker of aggressiveness, was found to be downregulated after PRIMA-1^MET^ exposure. While it was demonstrated that p53 negatively regulates CXCR4 in breast cancer [49], to our knowledge this modulation has never been reported in NB. Nevertheless, our result suggests that downregulation of CXCR4 could participate in NB growth inhibition induced by PRIMA-1^MET^.

## Conclusions

PRIMA-1^MET^ was identified as a potent and specific compound that inhibits growth in NB cell lines and kills cells rapidly by activation of apoptosis, autophagy, and oxidative stress. Although PRIMA-1^MET^ triggers p53 transactivation activity [18], its main targets remain inactive and inhibition of apoptosis is ineffective, suggesting an indirect interaction between PRIMA-1^MET^ and p53 in cases of NB. Importantly, PRIMA-1^MET^ is deactivated by free SH-groups, but it also causes a deep decrease in GSH concentration, through both direct binding and the inhibition of GSH synthesis. Finally, by showing that p53 and MYCN are involved in the modulation of resistance to PRIMA-1^MET^ through the modulation of intracellular GSH levels, we established a new model of the interaction between p53 and PRIMA-1^MET^. In the proposed model based on the presented results, p53 and MYCN induce PRIMA-1^MET^ resistance in an indirect manner, via GSH modulation, rather than via direct binding between PRIMA-1^MET^ and p53, which is the currently held view in many other cancer models (Fig. 6c).

## Additional files

Additional file 1: Whole exome sequencing of neuroblastoma cells. (XLSX 439 kb)

Additional file 2: Supplementary information for figures. (PPTX 6460 kb)

## Abbreviations

BSO: Buthionine sulfoximine
GSH: Glutathione,
IC50: 50% inhibitory concentration
ICW: In-cell western blot
LCL: Lymphoblastoid cell lines
MNA: MYCN amplification
MQ: Methylene quinuclidinone
NB: Neuroblastoma
r^2^: Correlation coefficient
SAH: S-adenosylhomocysteine
TXN: Thioredoxin
TXNRD1: Thioredoxin reductase 1
