# Supplementary material for: PRIMA-1MET-induced neuroblastoma cell death is modulated by p53 and mycn through glutathione level
Source: J Exp Clin Cancer Res. 2019 Feb 12;38:69. doi: 10.1186/s13046-019-1066-6 (PMC6373164; doi:10.1186/s13046-019-1066-6)
Supplement: Supplementary file 2 — Supplementary information for figures. (PPTX 6460 kb) [file 13046_2019_1066_MOESM2_ESM.pptx]

## Slide 1
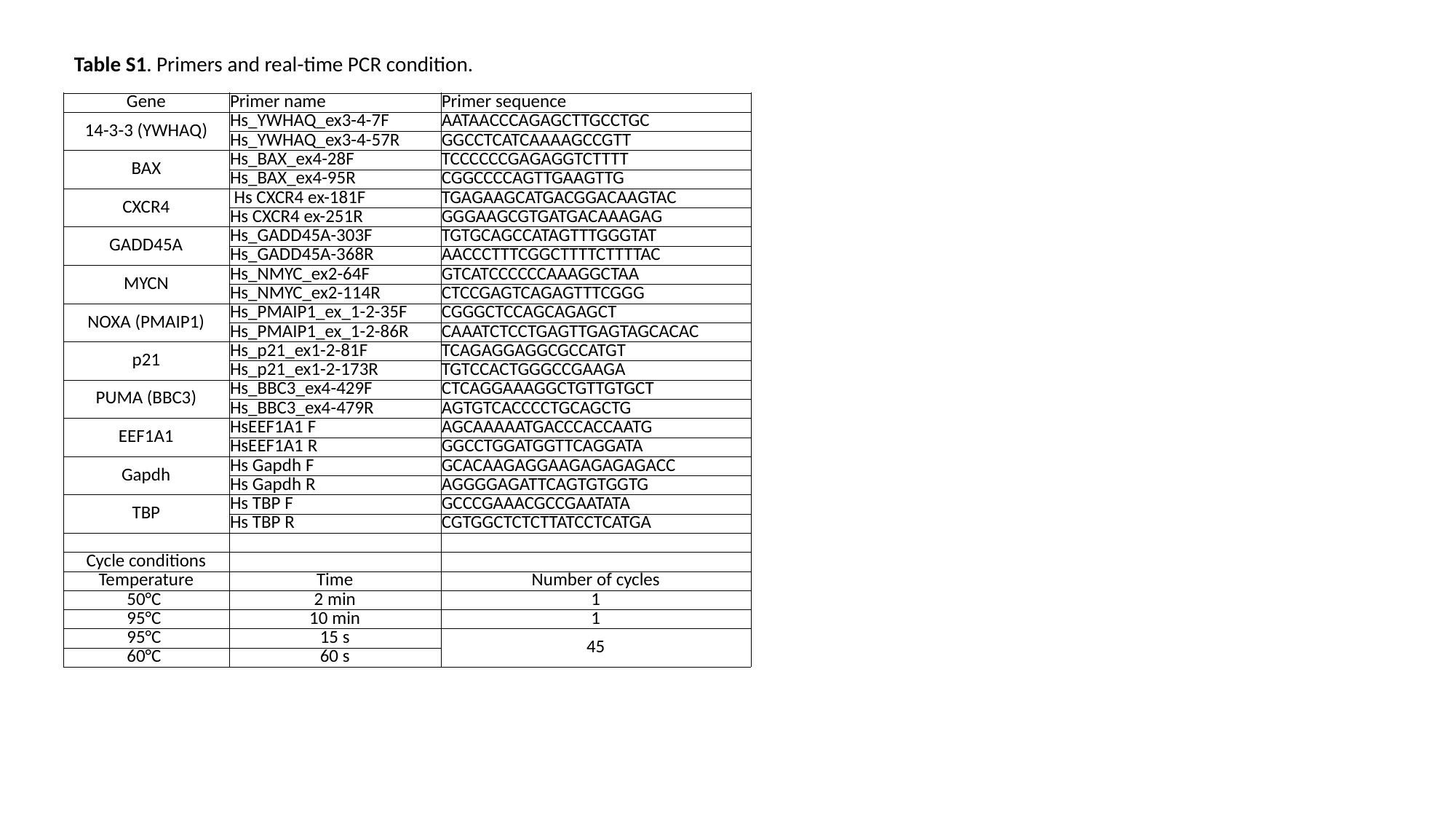

Table S1. Primers and real-time PCR condition.
| Gene | Primer name | Primer sequence |
| --- | --- | --- |
| 14-3-3 (YWHAQ) | Hs\_YWHAQ\_ex3-4-7F | AATAACCCAGAGCTTGCCTGC |
| | Hs\_YWHAQ\_ex3-4-57R | GGCCTCATCAAAAGCCGTT |
| BAX | Hs\_BAX\_ex4-28F | TCCCCCCGAGAGGTCTTTT |
| | Hs\_BAX\_ex4-95R | CGGCCCCAGTTGAAGTTG |
| CXCR4 | Hs CXCR4 ex-181F | TGAGAAGCATGACGGACAAGTAC |
| | Hs CXCR4 ex-251R | GGGAAGCGTGATGACAAAGAG |
| GADD45A | Hs\_GADD45A-303F | TGTGCAGCCATAGTTTGGGTAT |
| | Hs\_GADD45A-368R | AACCCTTTCGGCTTTTCTTTTAC |
| MYCN | Hs\_NMYC\_ex2-64F | GTCATCCCCCCAAAGGCTAA |
| | Hs\_NMYC\_ex2-114R | CTCCGAGTCAGAGTTTCGGG |
| NOXA (PMAIP1) | Hs\_PMAIP1\_ex\_1-2-35F | CGGGCTCCAGCAGAGCT |
| | Hs\_PMAIP1\_ex\_1-2-86R | CAAATCTCCTGAGTTGAGTAGCACAC |
| p21 | Hs\_p21\_ex1-2-81F | TCAGAGGAGGCGCCATGT |
| | Hs\_p21\_ex1-2-173R | TGTCCACTGGGCCGAAGA |
| PUMA (BBC3) | Hs\_BBC3\_ex4-429F | CTCAGGAAAGGCTGTTGTGCT |
| | Hs\_BBC3\_ex4-479R | AGTGTCACCCCTGCAGCTG |
| EEF1A1 | HsEEF1A1 F | AGCAAAAATGACCCACCAATG |
| | HsEEF1A1 R | GGCCTGGATGGTTCAGGATA |
| Gapdh | Hs Gapdh F | GCACAAGAGGAAGAGAGAGACC |
| | Hs Gapdh R | AGGGGAGATTCAGTGTGGTG |
| TBP | Hs TBP F | GCCCGAAACGCCGAATATA |
| | Hs TBP R | CGTGGCTCTCTTATCCTCATGA |
| | | |
| Cycle conditions | | |
| Temperature | Time | Number of cycles |
| 50°C | 2 min | 1 |
| 95°C | 10 min | 1 |
| 95°C | 15 s | 45 |
| 60°C | 60 s | |

## Slide 2
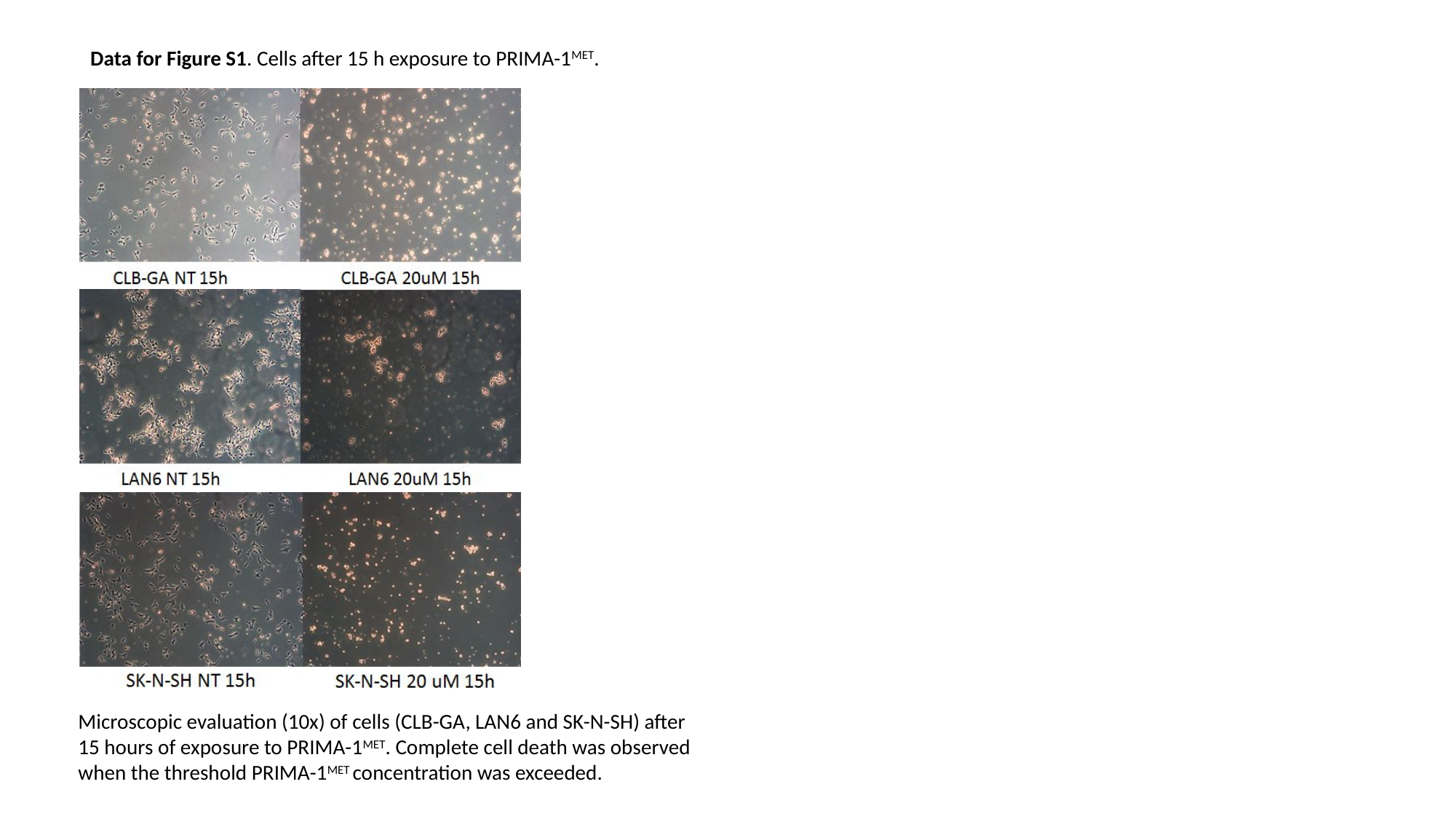

Data for Figure S1. Cells after 15 h exposure to PRIMA-1MET.
Microscopic evaluation (10x) of cells (CLB-GA, LAN6 and SK-N-SH) after 15 hours of exposure to PRIMA-1MET. Complete cell death was observed when the threshold PRIMA-1MET concentration was exceeded.

## Slide 3
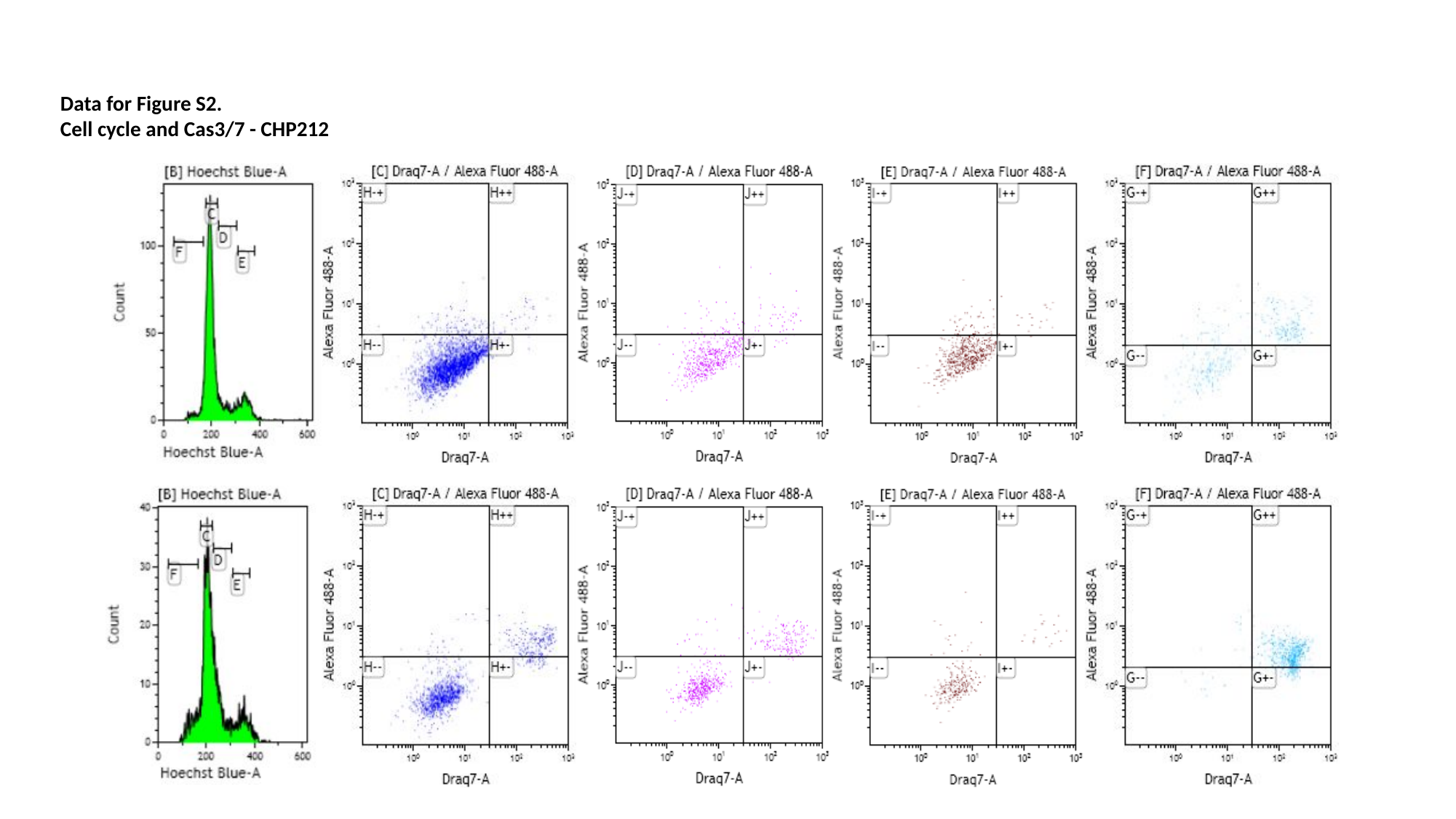

Data for Figure S2.
Cell cycle and Cas3/7 - CHP212

## Slide 4
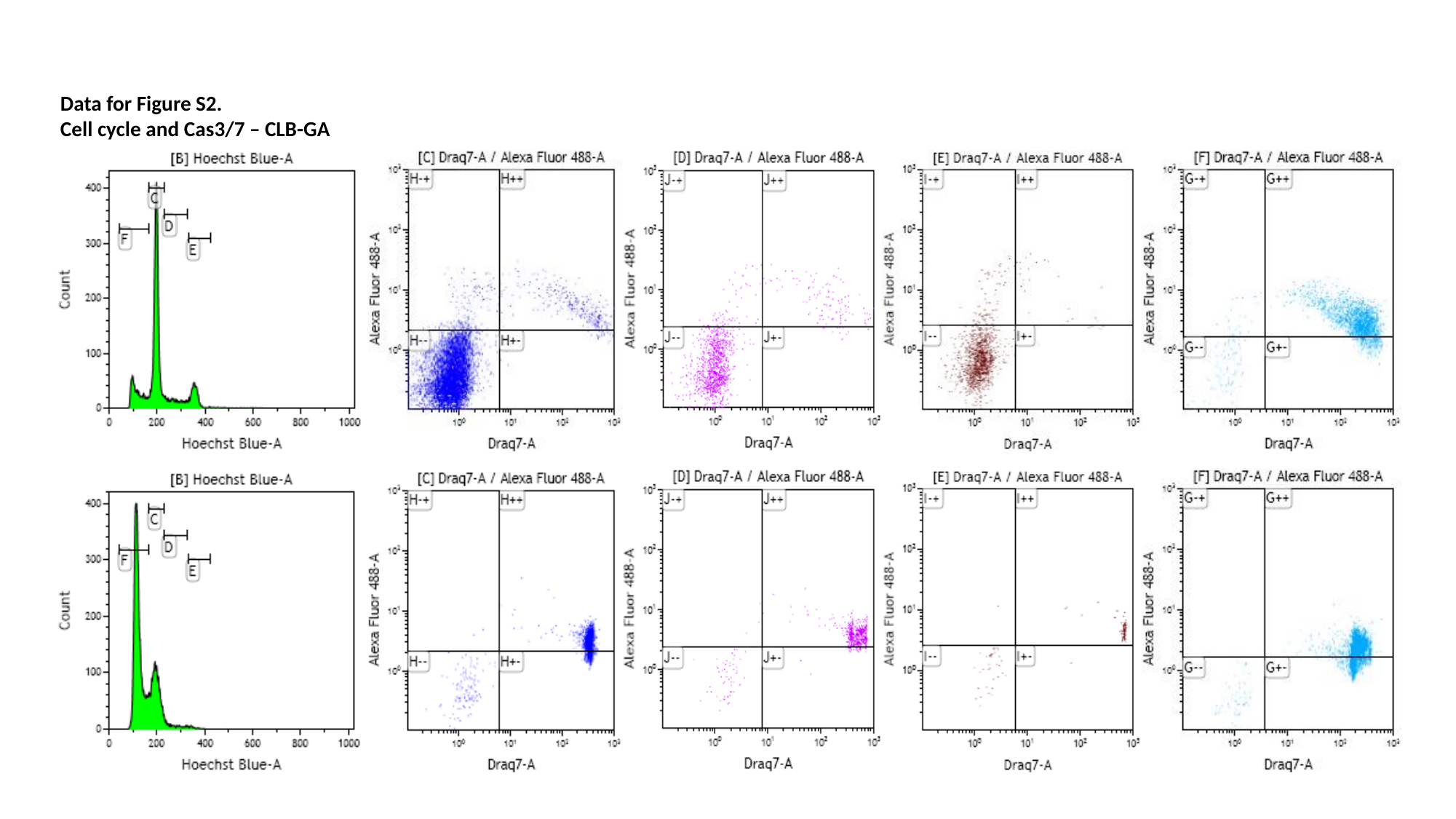

Data for Figure S2.
Cell cycle and Cas3/7 – CLB-GA

## Slide 5
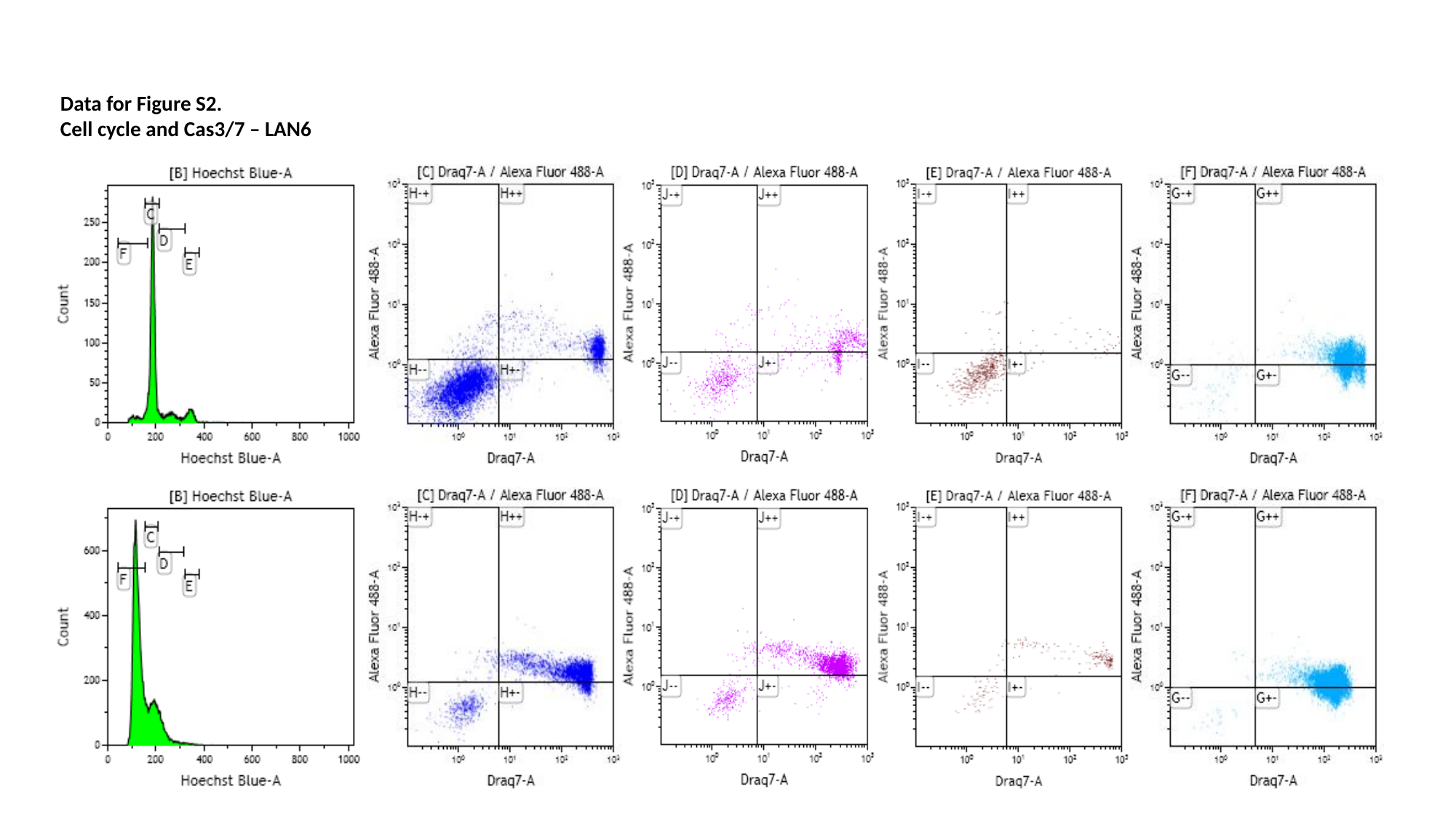

Data for Figure S2.
Cell cycle and Cas3/7 – LAN6

## Slide 6
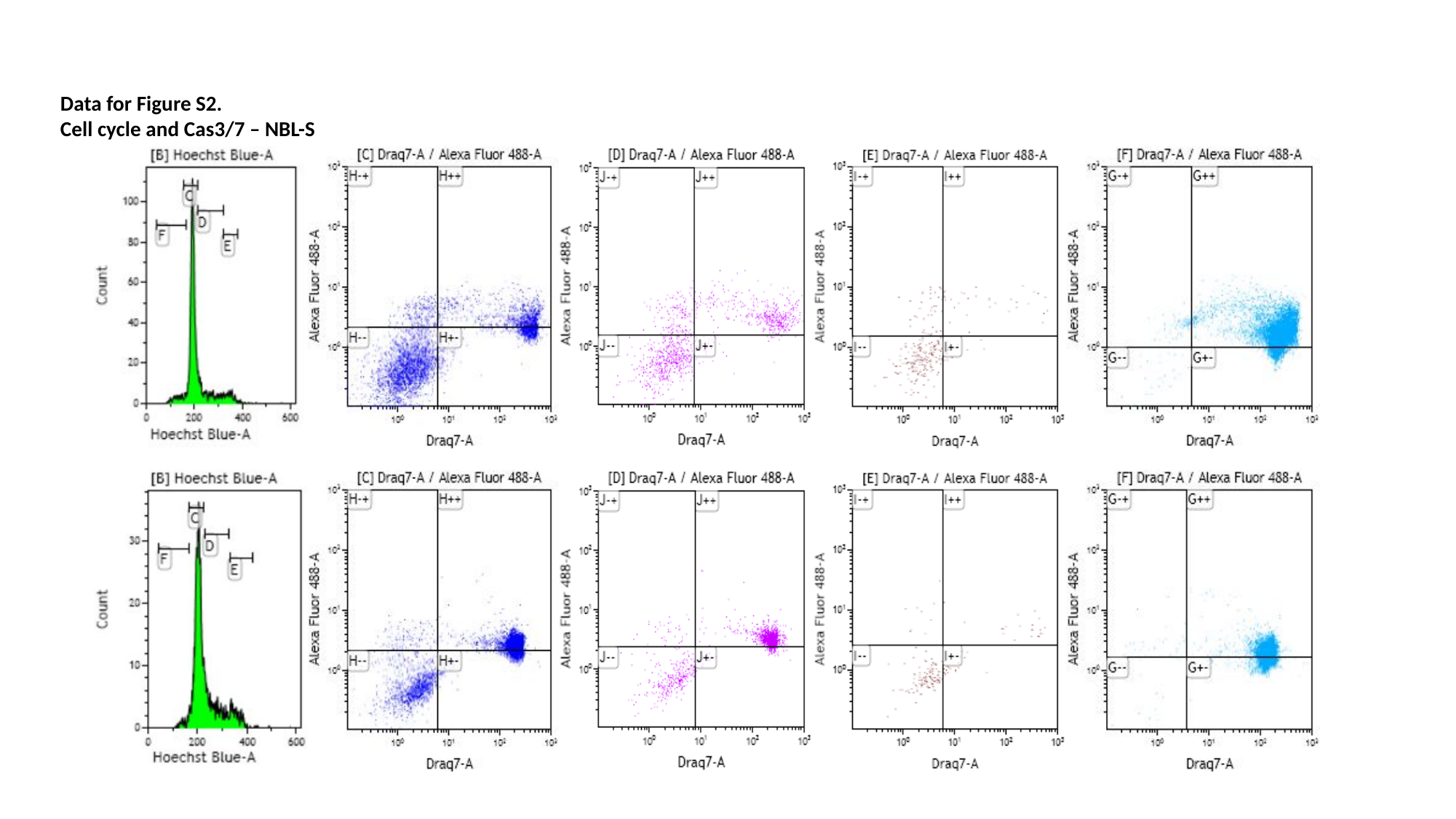

Data for Figure S2.
Cell cycle and Cas3/7 – NBL-S

## Slide 7
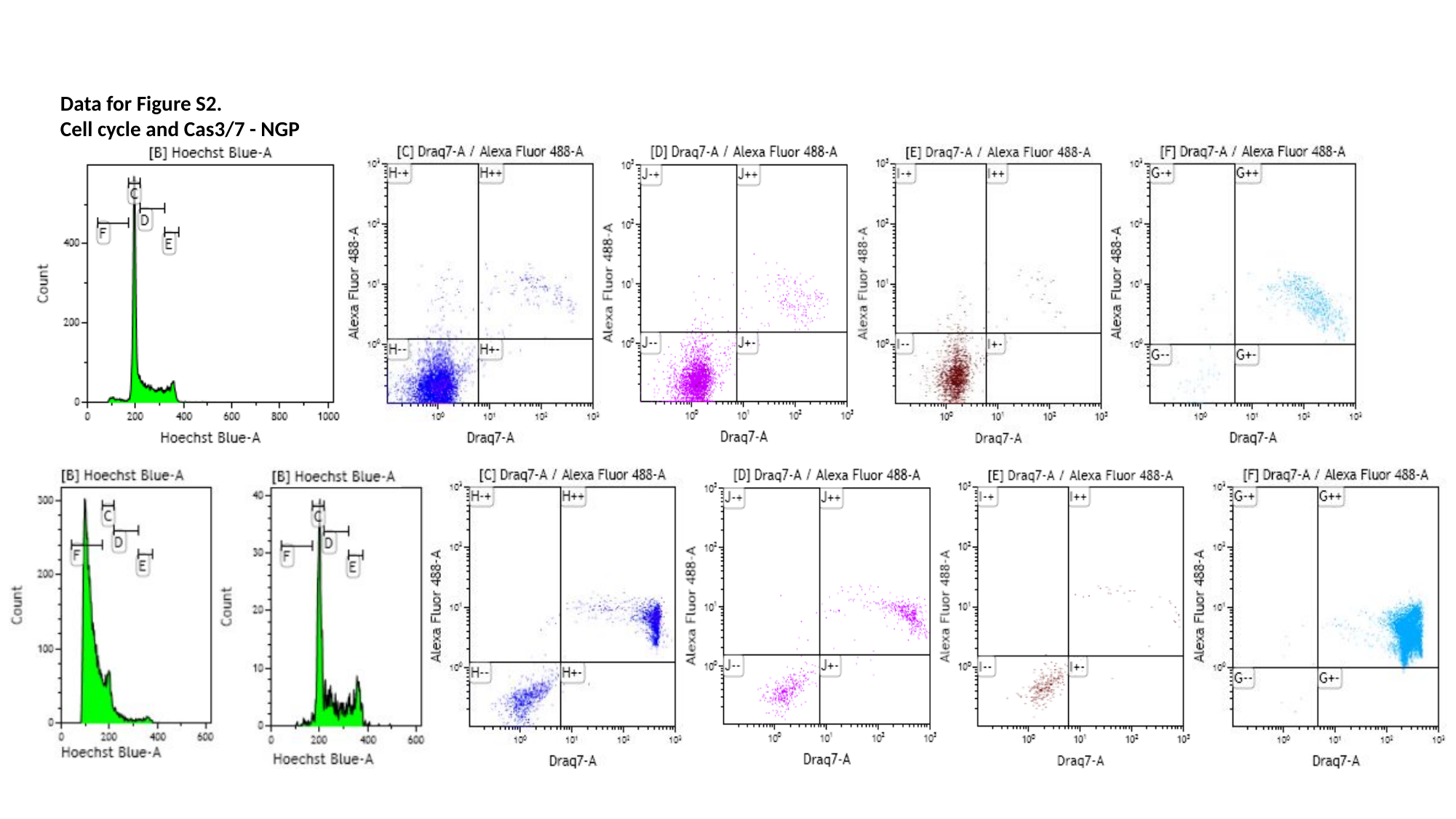

Data for Figure S2.
Cell cycle and Cas3/7 - NGP

## Slide 8
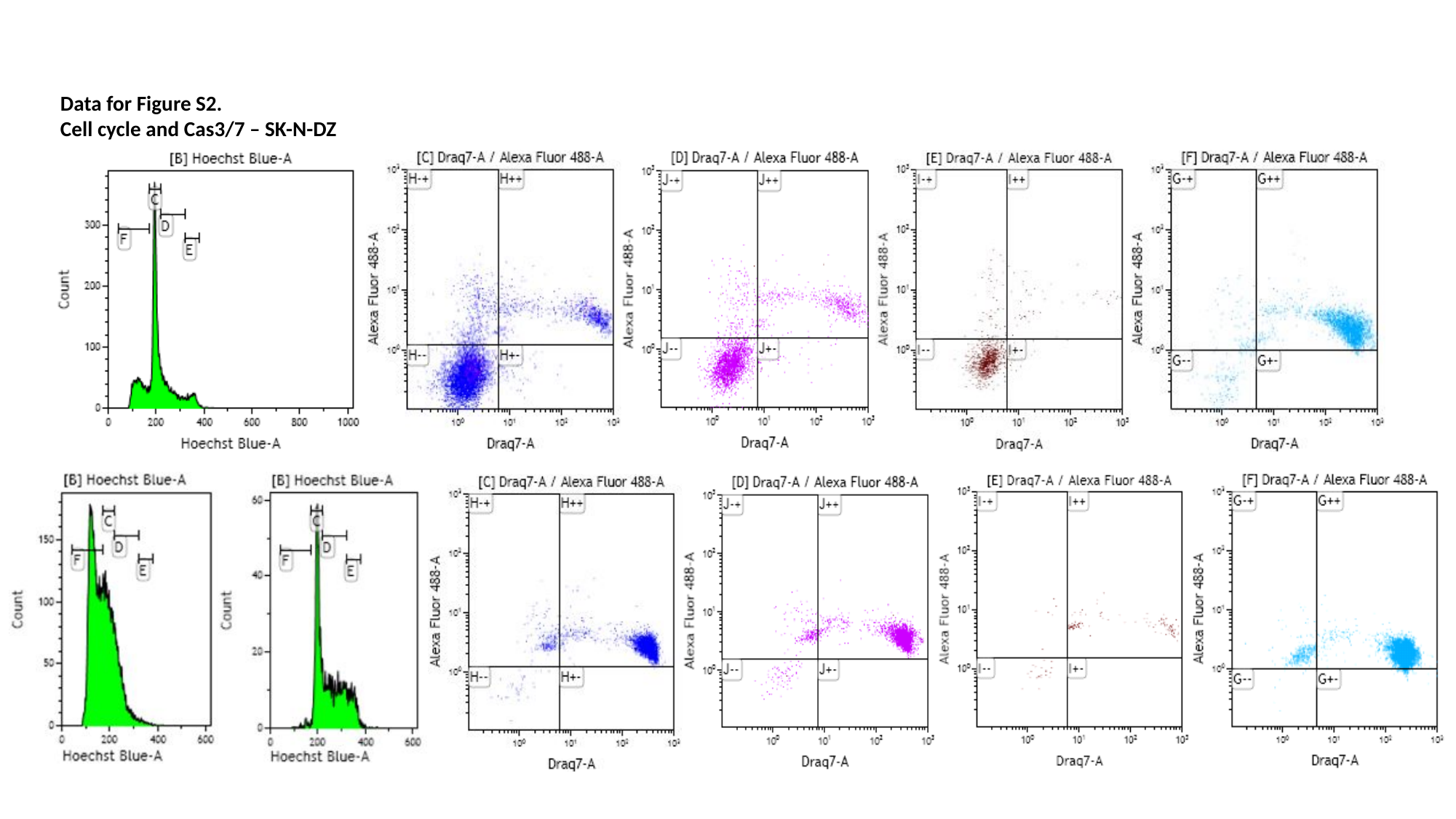

Data for Figure S2.
Cell cycle and Cas3/7 – SK-N-DZ

## Slide 9
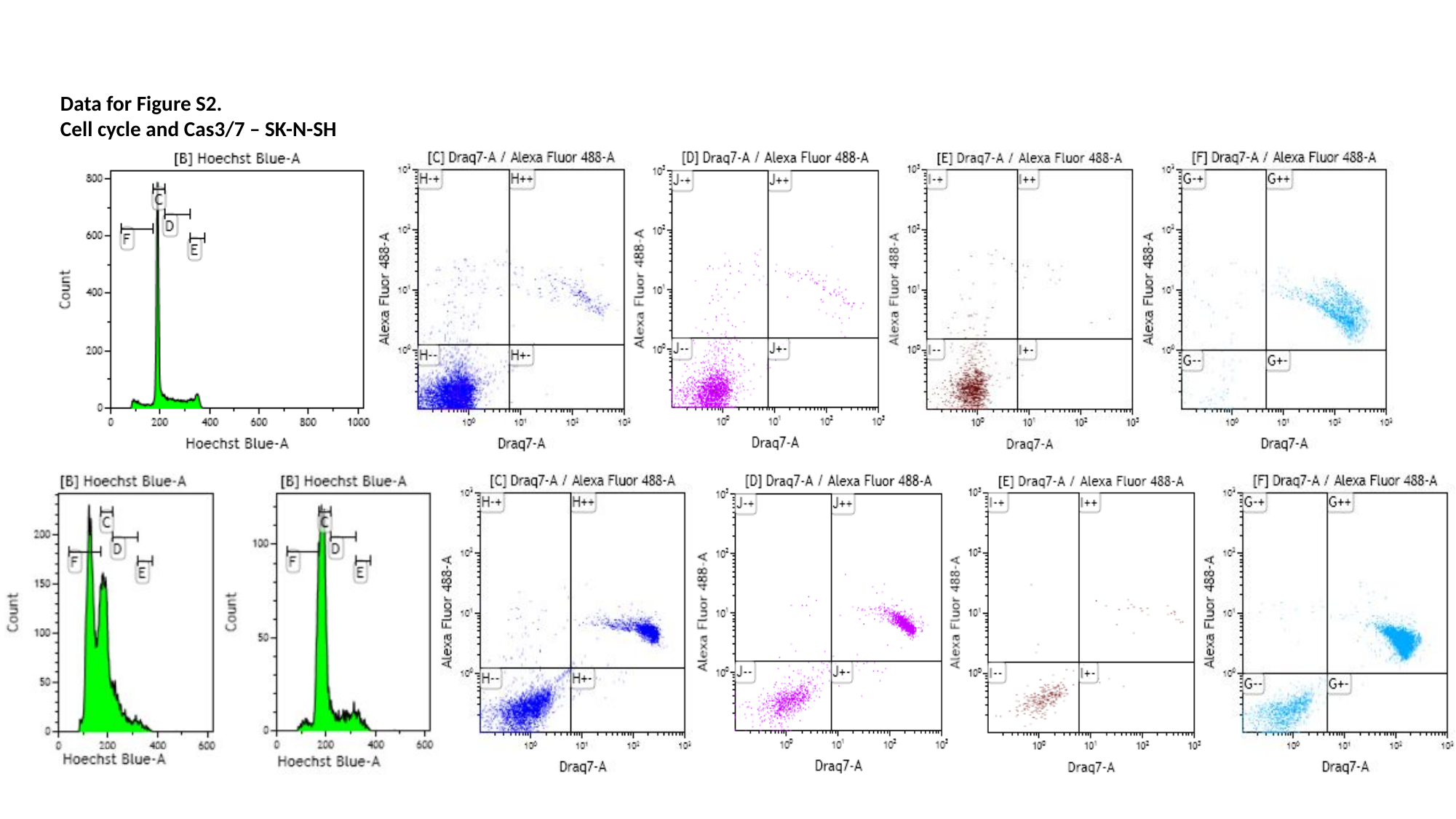

Data for Figure S2.
Cell cycle and Cas3/7 – SK-N-SH

## Slide 10
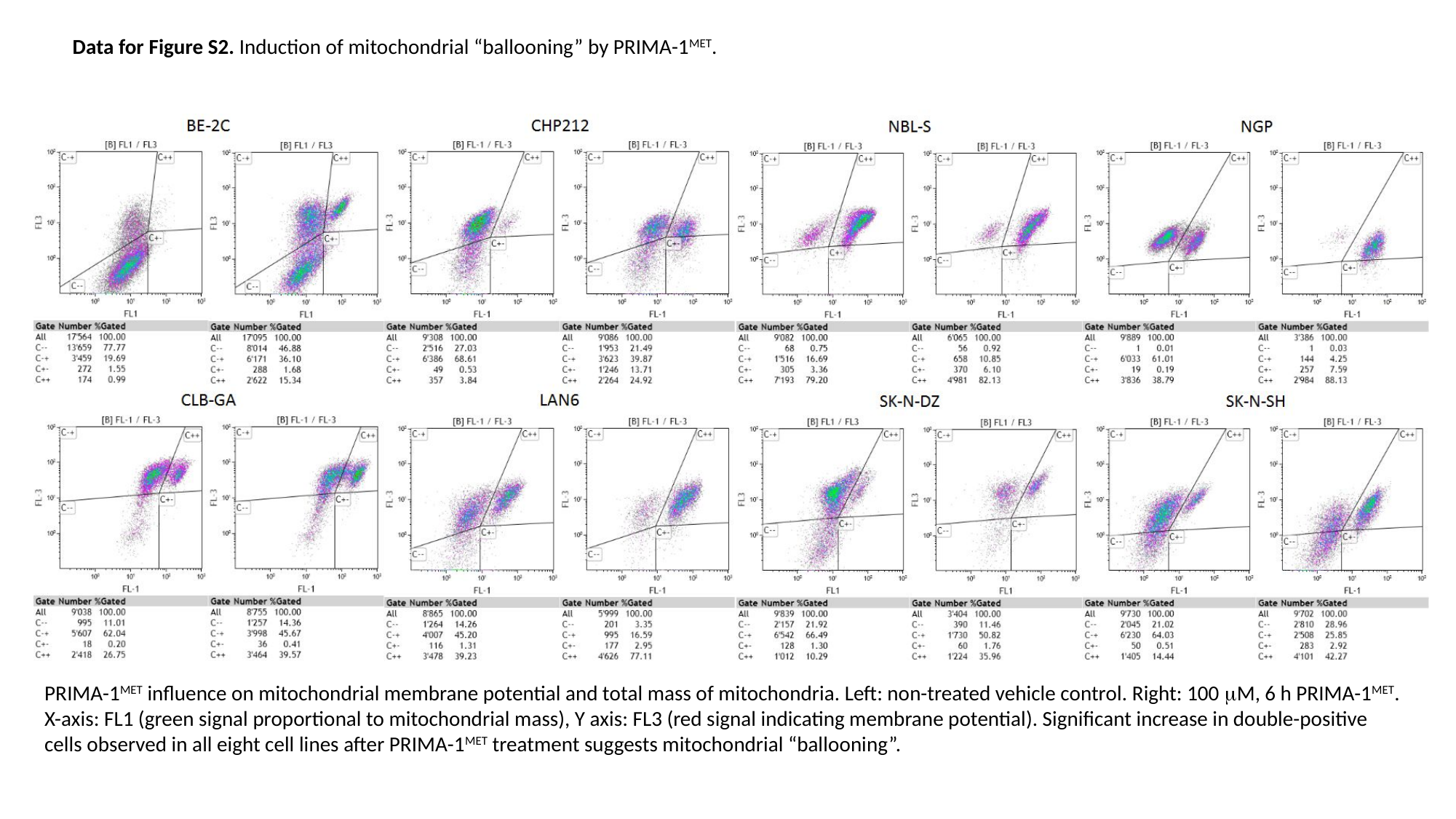

Data for Figure S2. Induction of mitochondrial “ballooning” by PRIMA-1MET.
PRIMA-1MET influence on mitochondrial membrane potential and total mass of mitochondria. Left: non-treated vehicle control. Right: 100 mM, 6 h PRIMA-1MET. X-axis: FL1 (green signal proportional to mitochondrial mass), Y axis: FL3 (red signal indicating membrane potential). Significant increase in double-positive cells observed in all eight cell lines after PRIMA-1MET treatment suggests mitochondrial “ballooning”.

## Slide 11
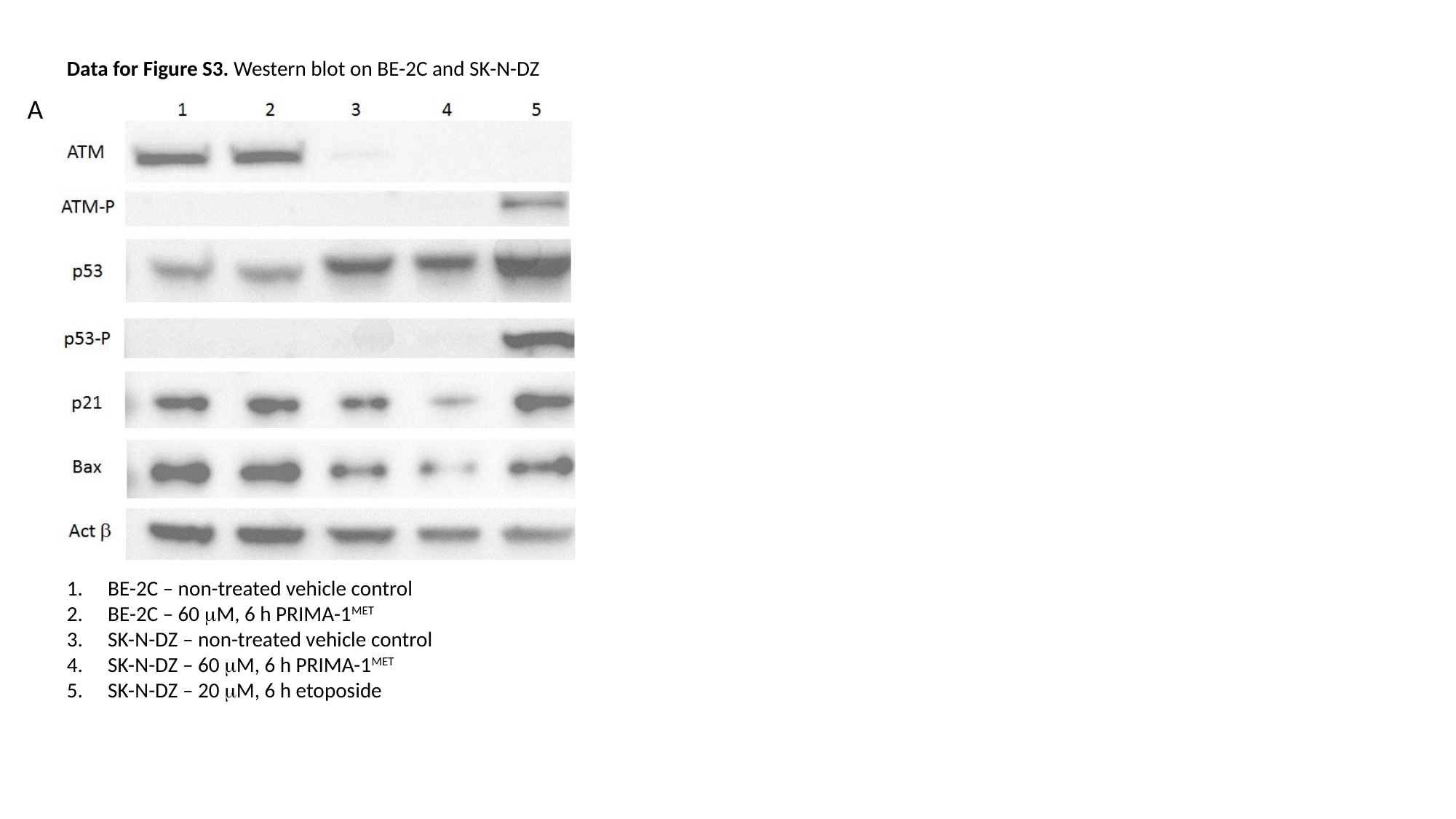

Data for Figure S3. Western blot on BE-2C and SK-N-DZ
A
BE-2C – non-treated vehicle control
BE-2C – 60 mM, 6 h PRIMA-1MET
SK-N-DZ – non-treated vehicle control
SK-N-DZ – 60 mM, 6 h PRIMA-1MET
SK-N-DZ – 20 mM, 6 h etoposide

## Slide 12
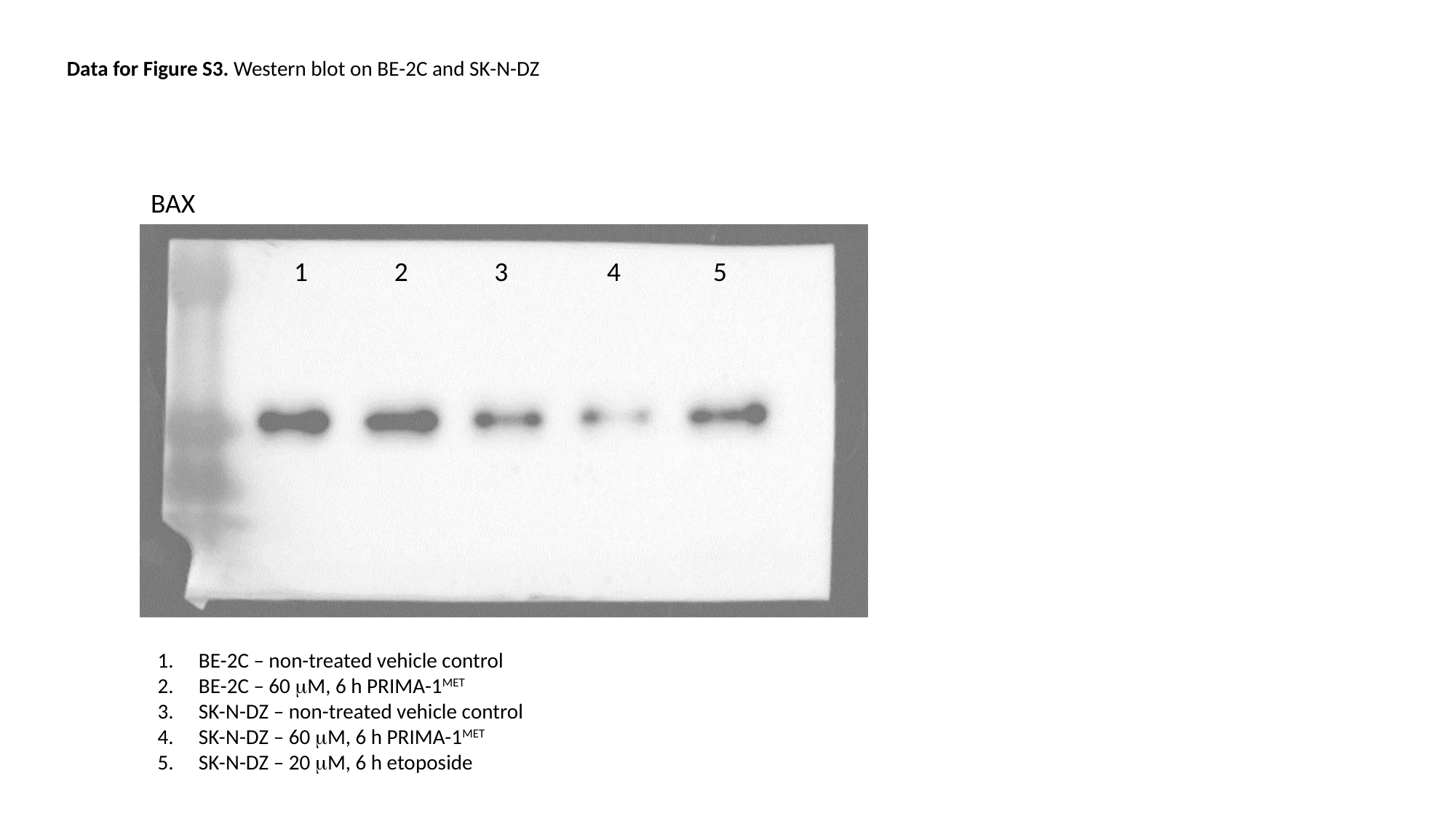

Data for Figure S3. Western blot on BE-2C and SK-N-DZ
BAX
1 2 3 4 5
BE-2C – non-treated vehicle control
BE-2C – 60 mM, 6 h PRIMA-1MET
SK-N-DZ – non-treated vehicle control
SK-N-DZ – 60 mM, 6 h PRIMA-1MET
SK-N-DZ – 20 mM, 6 h etoposide

## Slide 13
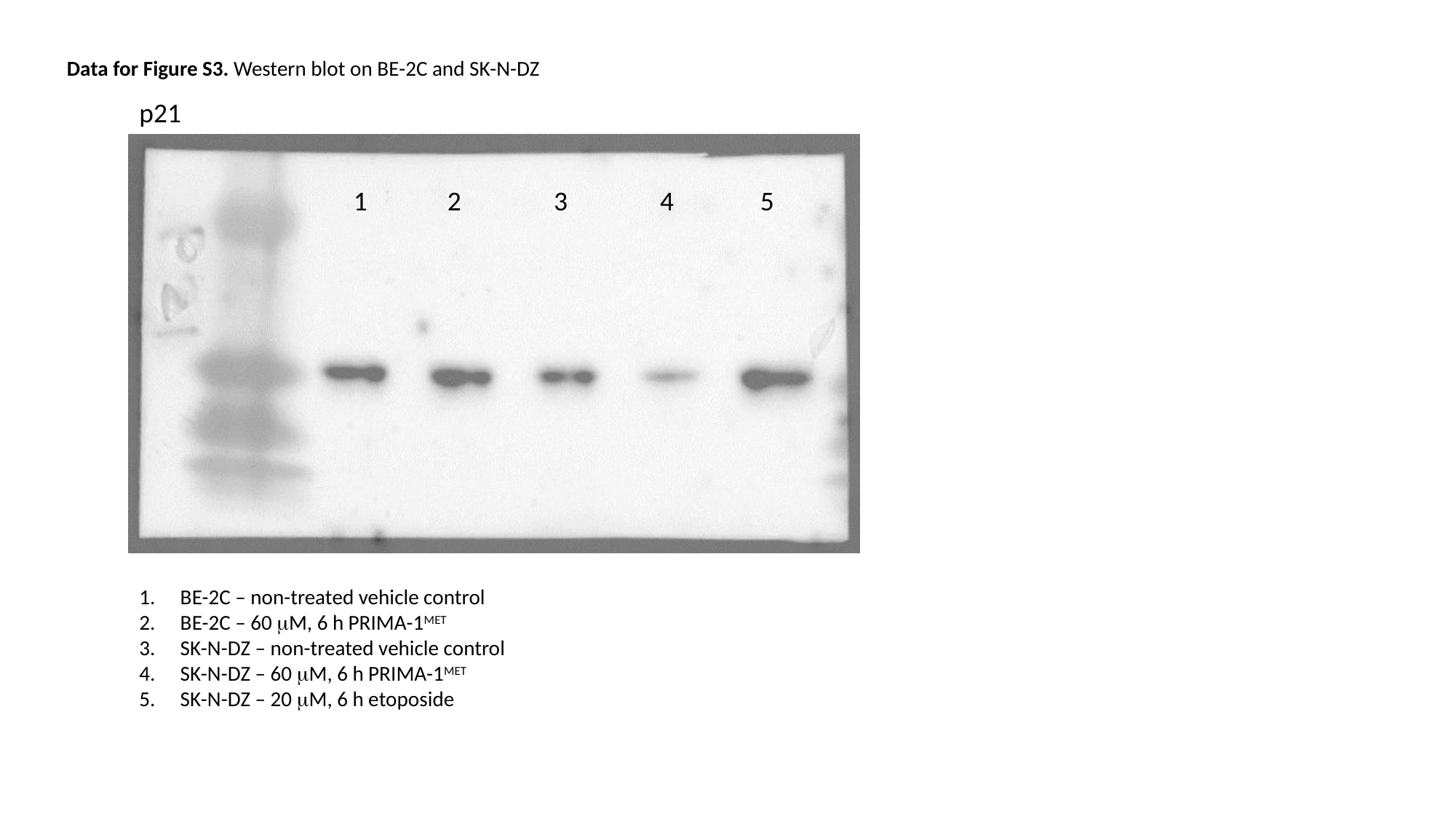

Data for Figure S3. Western blot on BE-2C and SK-N-DZ
p21
1 2 3 4 5
BE-2C – non-treated vehicle control
BE-2C – 60 mM, 6 h PRIMA-1MET
SK-N-DZ – non-treated vehicle control
SK-N-DZ – 60 mM, 6 h PRIMA-1MET
SK-N-DZ – 20 mM, 6 h etoposide

## Slide 14
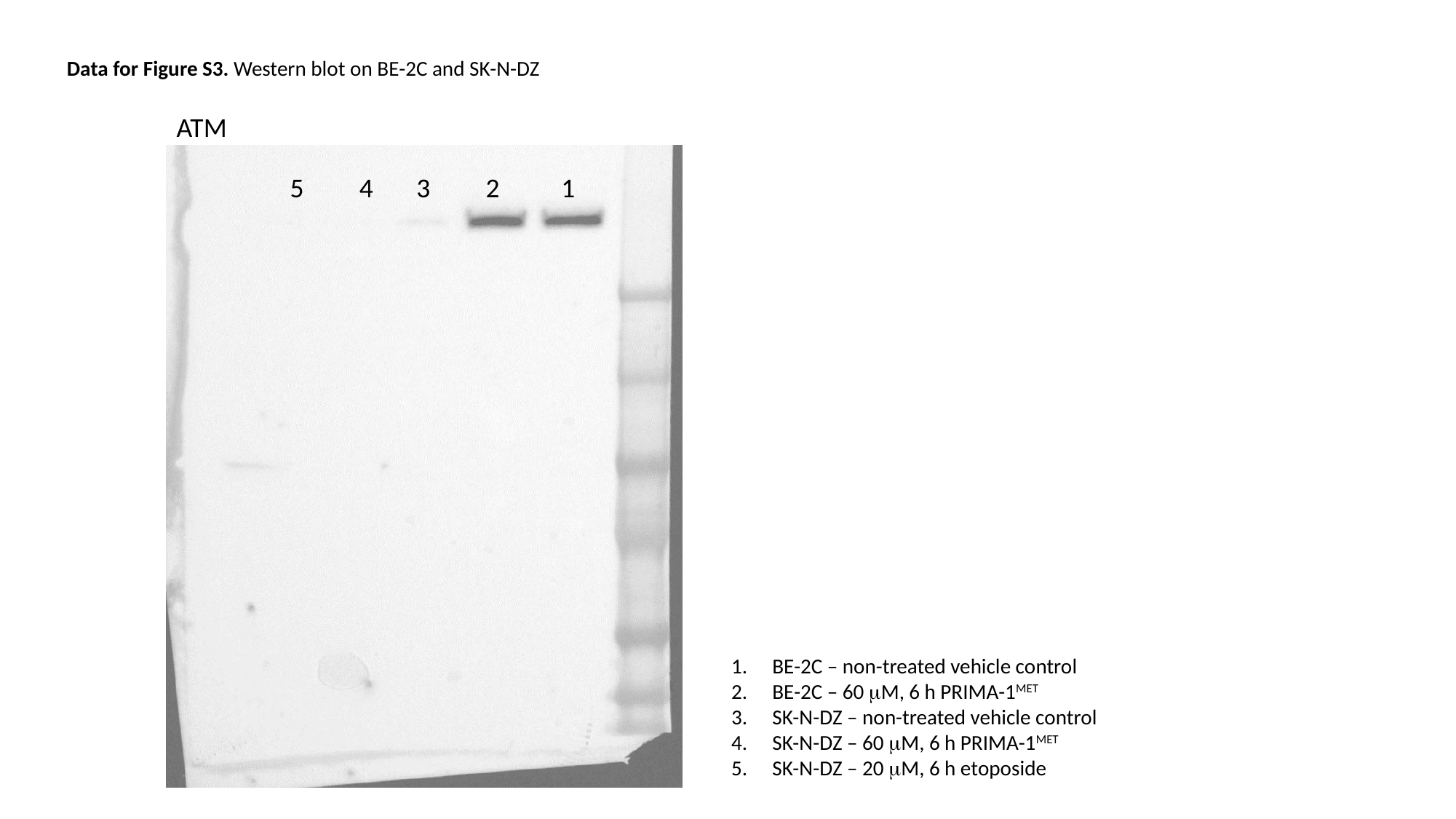

Data for Figure S3. Western blot on BE-2C and SK-N-DZ
ATM
5 4 3 2 1
BE-2C – non-treated vehicle control
BE-2C – 60 mM, 6 h PRIMA-1MET
SK-N-DZ – non-treated vehicle control
SK-N-DZ – 60 mM, 6 h PRIMA-1MET
SK-N-DZ – 20 mM, 6 h etoposide

## Slide 15
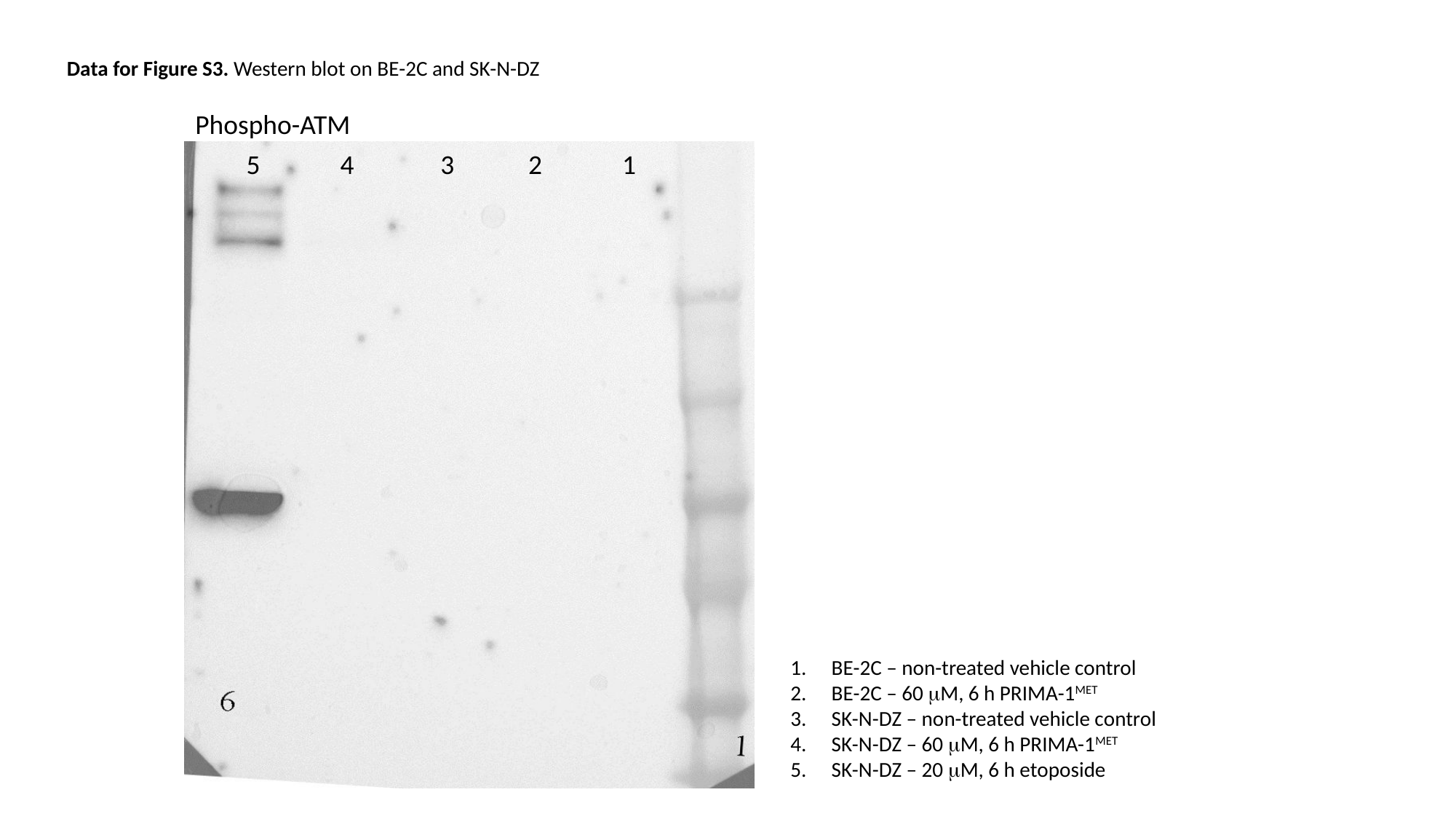

Data for Figure S3. Western blot on BE-2C and SK-N-DZ
Phospho-ATM
5 4 3 2 1
BE-2C – non-treated vehicle control
BE-2C – 60 mM, 6 h PRIMA-1MET
SK-N-DZ – non-treated vehicle control
SK-N-DZ – 60 mM, 6 h PRIMA-1MET
SK-N-DZ – 20 mM, 6 h etoposide

## Slide 16
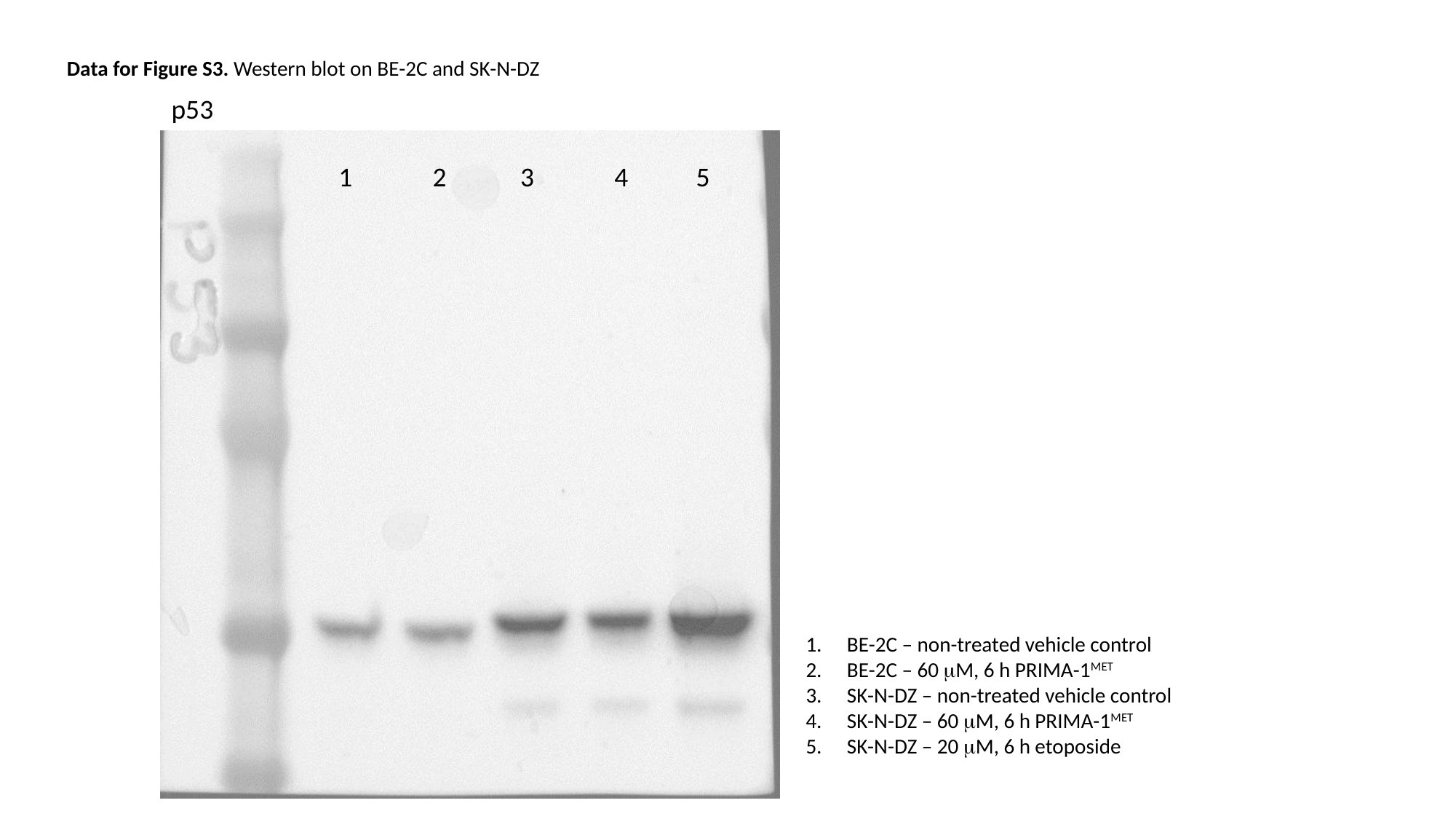

Data for Figure S3. Western blot on BE-2C and SK-N-DZ
p53
1 2 3 4 5
BE-2C – non-treated vehicle control
BE-2C – 60 mM, 6 h PRIMA-1MET
SK-N-DZ – non-treated vehicle control
SK-N-DZ – 60 mM, 6 h PRIMA-1MET
SK-N-DZ – 20 mM, 6 h etoposide

## Slide 17
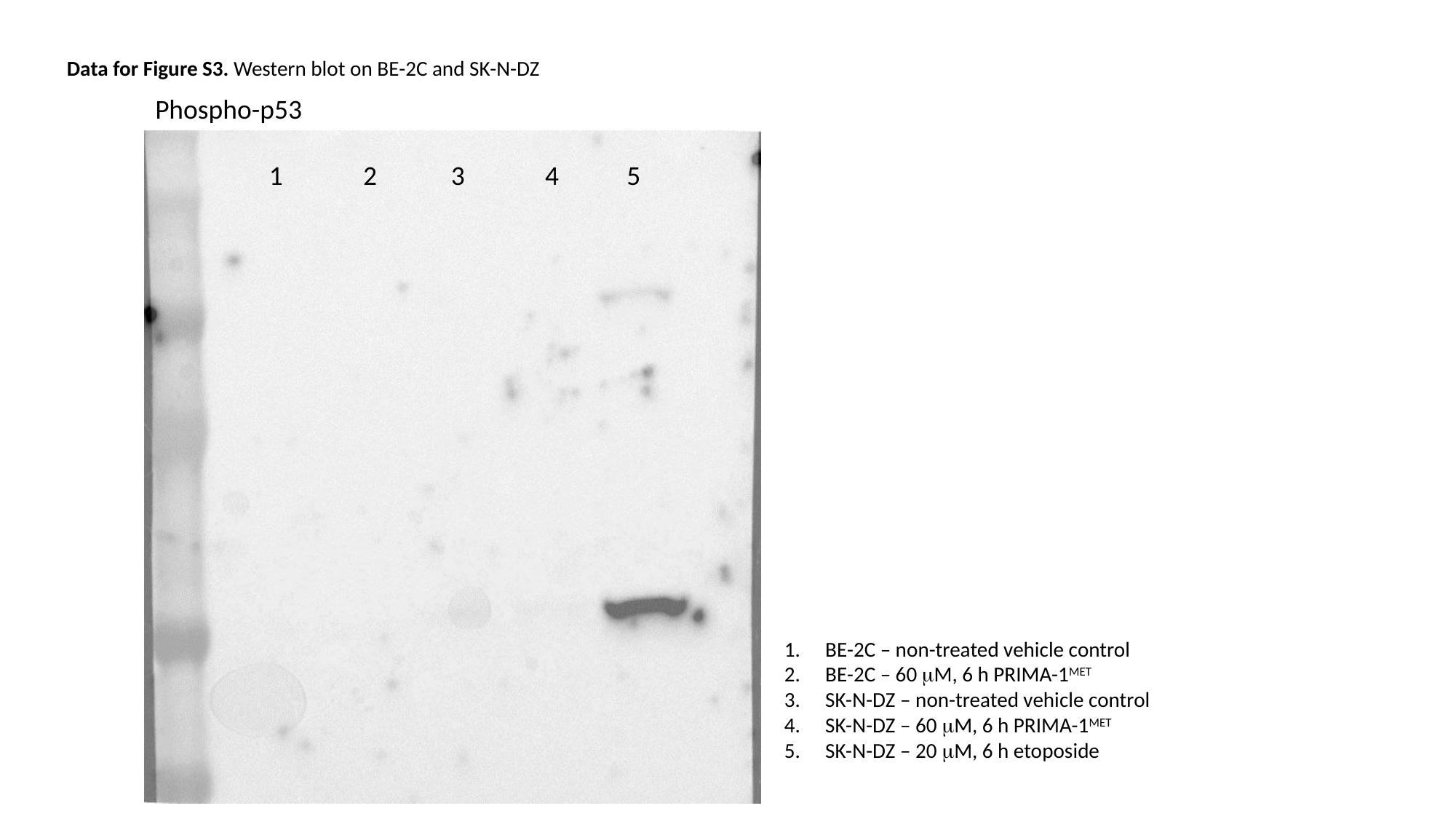

Data for Figure S3. Western blot on BE-2C and SK-N-DZ
Phospho-p53
1 2 3 4 5
BE-2C – non-treated vehicle control
BE-2C – 60 mM, 6 h PRIMA-1MET
SK-N-DZ – non-treated vehicle control
SK-N-DZ – 60 mM, 6 h PRIMA-1MET
SK-N-DZ – 20 mM, 6 h etoposide

## Slide 18
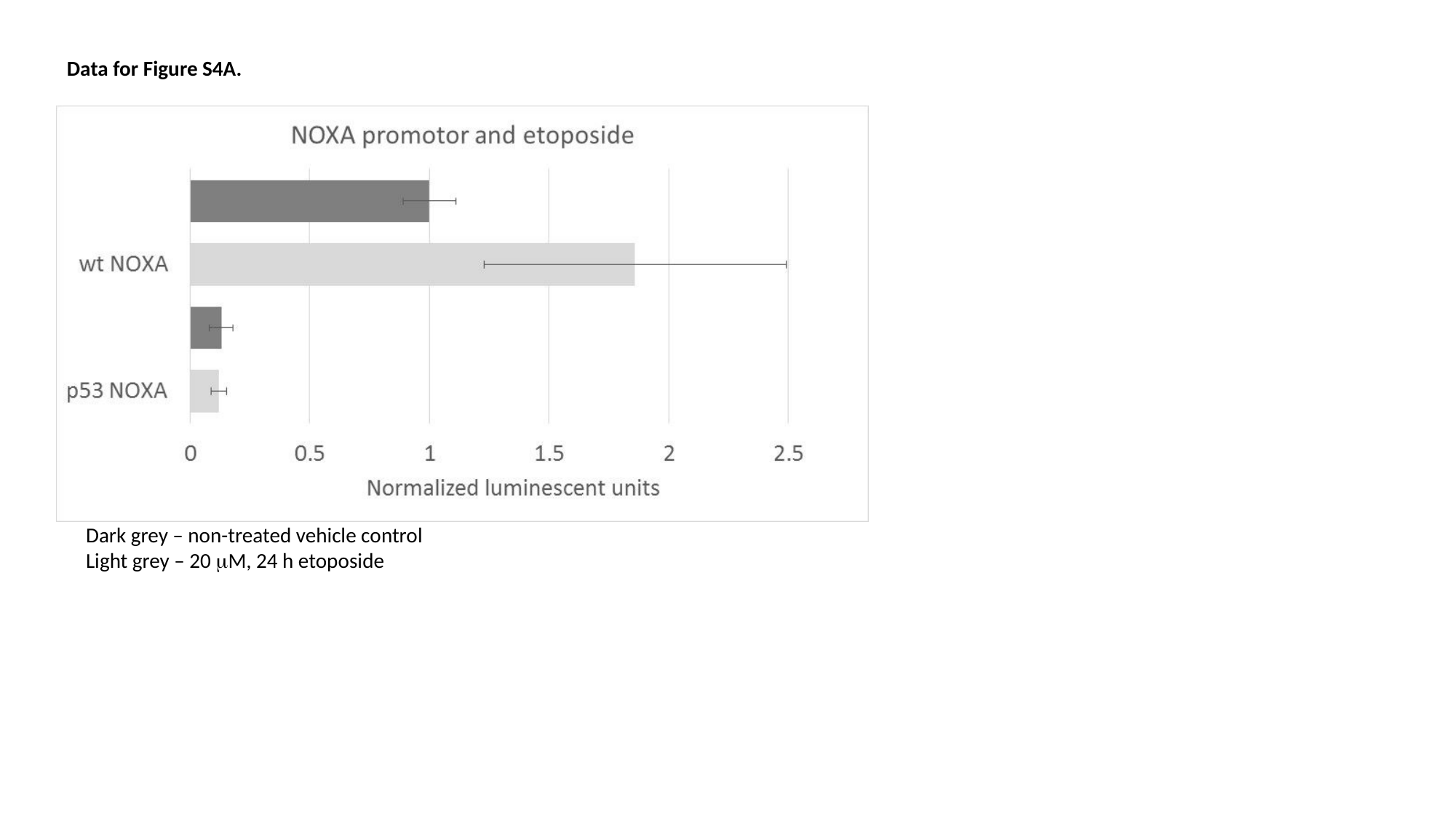

Data for Figure S4A.
Dark grey – non-treated vehicle control
Light grey – 20 mM, 24 h etoposide

## Slide 19
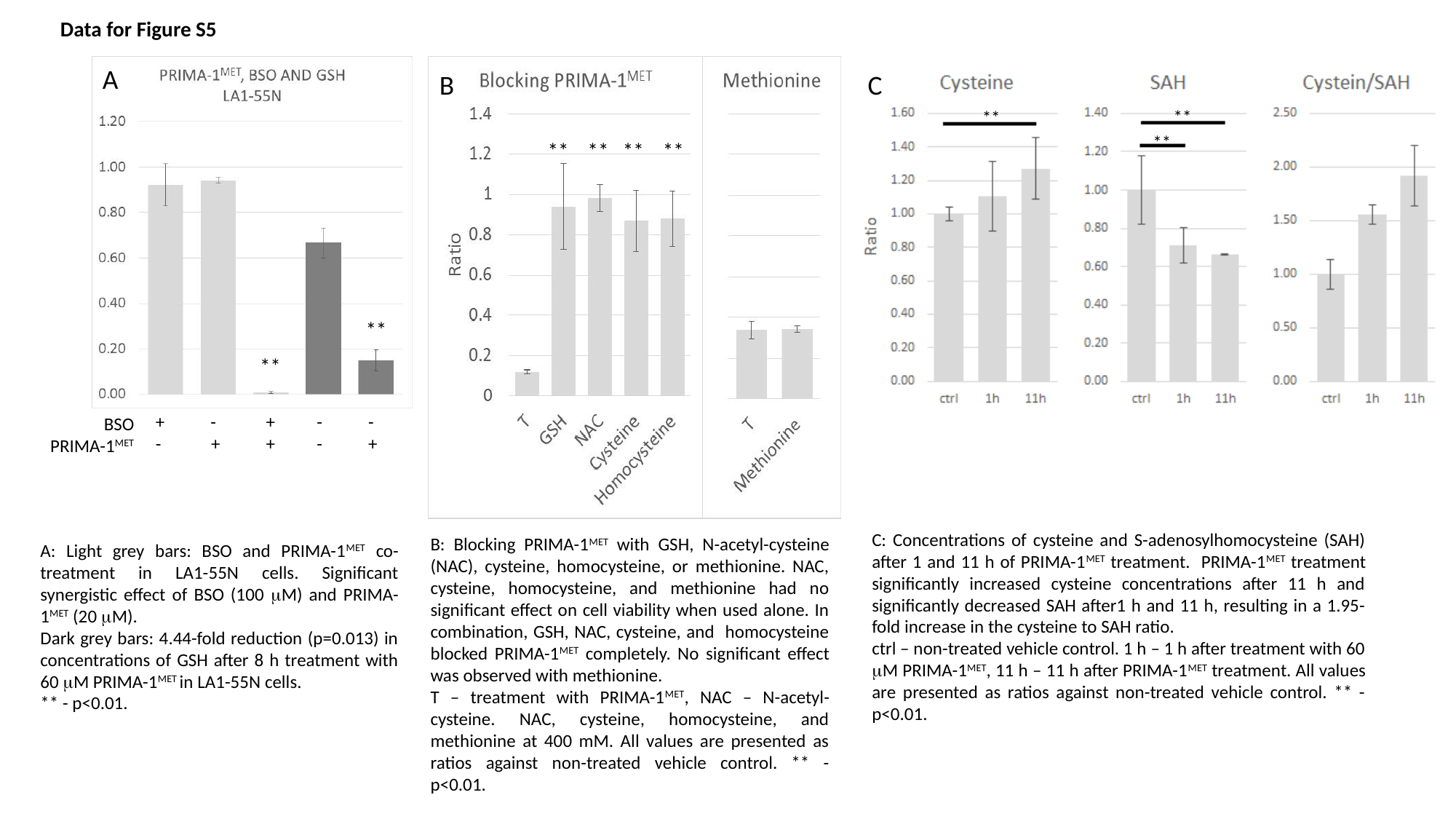

Data for Figure S5
A
+ - + - -
- + + - +
BSO
PRIMA-1MET
** ** ** **
B
C
**
**
**
**
**
C: Concentrations of cysteine and S-adenosylhomocysteine (SAH) after 1 and 11 h of PRIMA-1MET treatment. PRIMA-1MET treatment significantly increased cysteine concentrations after 11 h and significantly decreased SAH after1 h and 11 h, resulting in a 1.95-fold increase in the cysteine to SAH ratio.
ctrl – non-treated vehicle control. 1 h – 1 h after treatment with 60 mM PRIMA-1MET, 11 h – 11 h after PRIMA-1MET treatment. All values are presented as ratios against non-treated vehicle control. ** - p<0.01.
B: Blocking PRIMA-1MET with GSH, N-acetyl-cysteine (NAC), cysteine, homocysteine, or methionine. NAC, cysteine, homocysteine, and methionine had no significant effect on cell viability when used alone. In combination, GSH, NAC, cysteine, and homocysteine blocked PRIMA-1MET completely. No significant effect was observed with methionine.
T – treatment with PRIMA-1MET, NAC – N-acetyl-cysteine. NAC, cysteine, homocysteine, and methionine at 400 mM. All values are presented as ratios against non-treated vehicle control. ** - p<0.01.
A: Light grey bars: BSO and PRIMA-1MET co-treatment in LA1-55N cells. Significant synergistic effect of BSO (100 mM) and PRIMA-1MET (20 mM).
Dark grey bars: 4.44-fold reduction (p=0.013) in concentrations of GSH after 8 h treatment with 60 mM PRIMA-1MET in LA1-55N cells.
** - p<0.01.

## Slide 20
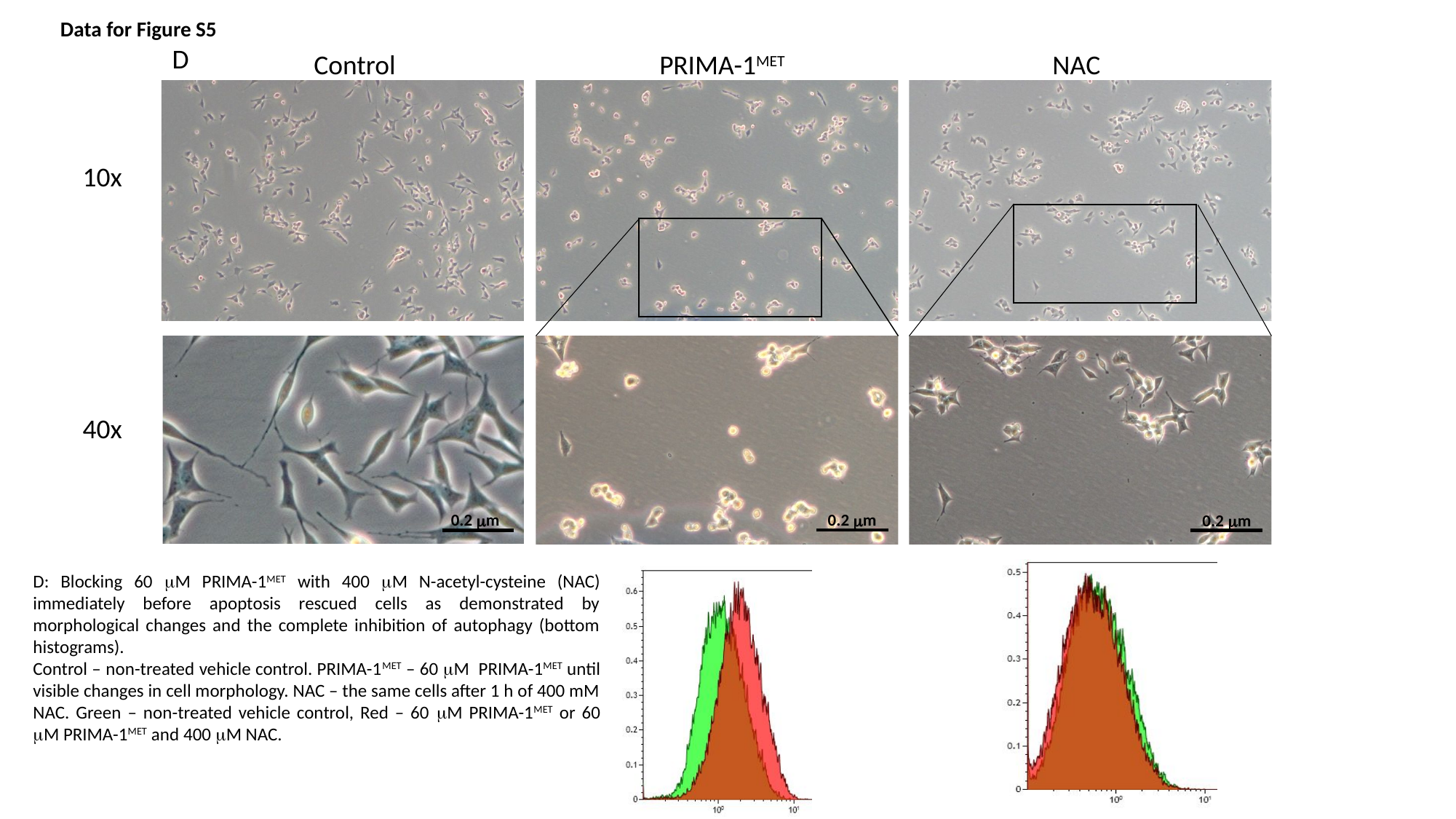

Data for Figure S5
D
Control
NAC
PRIMA-1MET
0.2 mm
0.2 mm
0.2 mm
10x
40x
D: Blocking 60 mM PRIMA-1MET with 400 mM N-acetyl-cysteine (NAC) immediately before apoptosis rescued cells as demonstrated by morphological changes and the complete inhibition of autophagy (bottom histograms).
Control – non-treated vehicle control. PRIMA-1MET – 60 mM PRIMA-1MET until visible changes in cell morphology. NAC – the same cells after 1 h of 400 mM NAC. Green – non-treated vehicle control, Red – 60 mM PRIMA-1MET or 60 mM PRIMA-1MET and 400 mM NAC.

## Slide 21
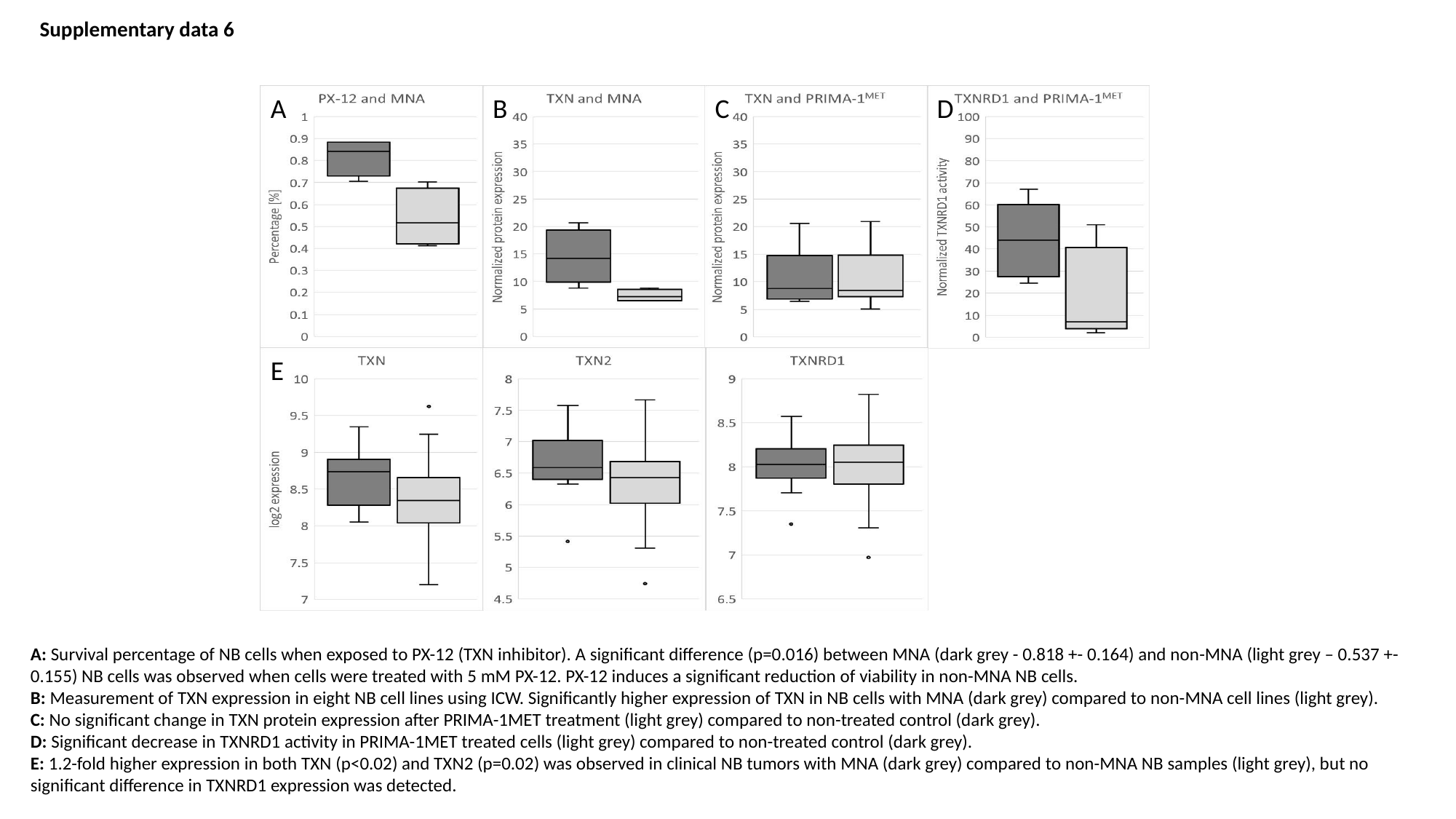

Supplementary data 6
A
B
C
D
E
A: Survival percentage of NB cells when exposed to PX-12 (TXN inhibitor). A significant difference (p=0.016) between MNA (dark grey - 0.818 +- 0.164) and non-MNA (light grey – 0.537 +- 0.155) NB cells was observed when cells were treated with 5 mM PX-12. PX-12 induces a significant reduction of viability in non-MNA NB cells.
B: Measurement of TXN expression in eight NB cell lines using ICW. Significantly higher expression of TXN in NB cells with MNA (dark grey) compared to non-MNA cell lines (light grey).
C: No significant change in TXN protein expression after PRIMA-1MET treatment (light grey) compared to non-treated control (dark grey).
D: Significant decrease in TXNRD1 activity in PRIMA-1MET treated cells (light grey) compared to non-treated control (dark grey).
E: 1.2-fold higher expression in both TXN (p<0.02) and TXN2 (p=0.02) was observed in clinical NB tumors with MNA (dark grey) compared to non-MNA NB samples (light grey), but no significant difference in TXNRD1 expression was detected.

## Slide 22
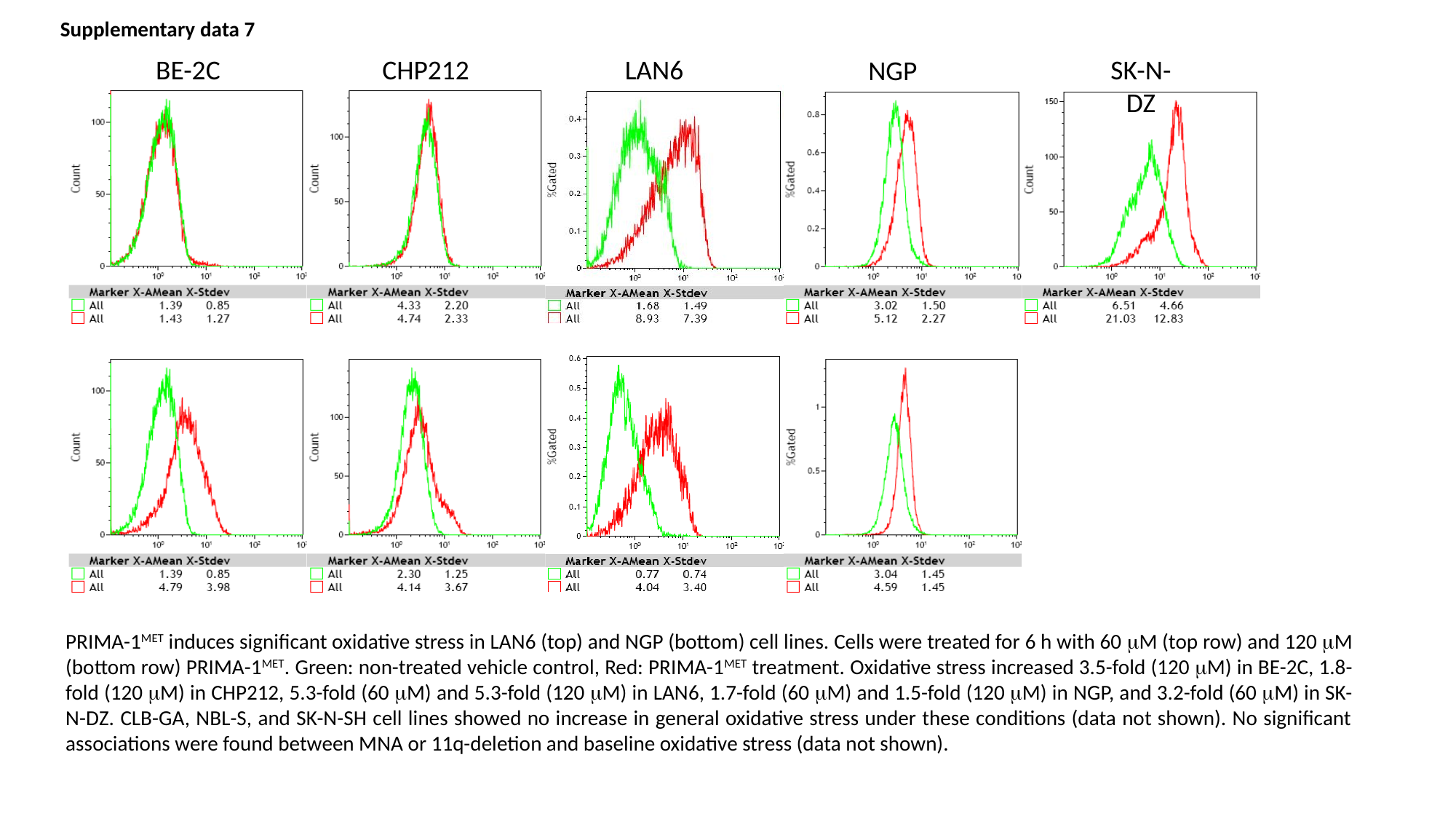

Supplementary data 7
BE-2C
LAN6
SK-N-DZ
CHP212
NGP
PRIMA-1MET induces significant oxidative stress in LAN6 (top) and NGP (bottom) cell lines. Cells were treated for 6 h with 60 mM (top row) and 120 mM (bottom row) PRIMA-1MET. Green: non-treated vehicle control, Red: PRIMA-1MET treatment. Oxidative stress increased 3.5-fold (120 mM) in BE-2C, 1.8-fold (120 mM) in CHP212, 5.3-fold (60 mM) and 5.3-fold (120 mM) in LAN6, 1.7-fold (60 mM) and 1.5-fold (120 mM) in NGP, and 3.2-fold (60 mM) in SK-N-DZ. CLB-GA, NBL-S, and SK-N-SH cell lines showed no increase in general oxidative stress under these conditions (data not shown). No significant associations were found between MNA or 11q-deletion and baseline oxidative stress (data not shown).
